# Pediatric complex chronic conditions classification system version 2: updated for ICD-10 and complex medical technology dependence and transplantation

**DOI:** 10.1186/1471-2431-14-199

**Published:** 2014-08-08

**Authors:** Chris Feudtner, James A Feinstein, Wenjun Zhong, Matt Hall, Dingwei Dai

**Affiliations:** 1 Pediatric Advanced Care Team and the Center for Pediatric Clinical Effectiveness, The Children’s Hospital of Philadelphia, CHOP North-Room 1523, 34th and Civic Center Blvd, Philadelphia, PA 10194, USA; 2 Department of Pediatrics, The Perelman School of Medicine at the University of Pennsylvania, Philadelphia, PA, USA; 3 The Department of Medical Ethics and Health Policy and the Leonard Davis Institute, University of Pennsylvania, Philadelphia, PA, USA; 4 Children’s Outcomes Research Program, Children’s Hospital Colorado, Aurora, CO, USA; 5 Department of Pediatrics, University of Colorado School of Medicine, Aurora, CO, USA; 6 Children’s Hospital Association, Overland Park, KS, USA

**Keywords:** Pediatrics, Complex chronic conditions, Chronic disease, Classification, International classification of disease codes, Comorbidity, Mortality, Health services research

## Abstract

**Background:**

The pediatric complex chronic conditions (CCC) classification system, developed in 2000, requires revision to accommodate the International Classification of Disease 10th Revision (ICD-10). To update the CCC classification system, we incorporated ICD-9 diagnostic codes that had been either omitted or incorrectly specified in the original system, and then translated between ICD-9 and ICD-10 using General Equivalence Mappings (GEMs). We further reviewed all codes in the ICD-9 and ICD-10 systems to include both diagnostic and procedural codes indicative of technology dependence or organ transplantation. We applied the provisional CCC version 2 (v2) system to death certificate information and 2 databases of health utilization, reviewed the resulting CCC classifications, and corrected any misclassifications. Finally, we evaluated performance of the CCC v2 system by assessing: 1) the stability of the system between ICD-9 and ICD-10 codes using data which included both ICD-9 codes and ICD-10 codes; 2) the year-to-year stability before and after ICD-10 implementation; and 3) the proportions of patients classified as having a CCC in both the v1 and v2 systems.

**Results:**

The CCC v2 classification system consists of diagnostic and procedural codes that incorporate a new neonatal CCC category as well as domains of complexity arising from technology dependence or organ transplantation. CCC v2 demonstrated close comparability between ICD-9 and ICD-10 and did not detect significant discontinuity in temporal trends of death in the United States. Compared to the original system, CCC v2 resulted in a 1.0% absolute (10% relative) increase in the number of patients identified as having a CCC in national hospitalization dataset, and a 0.4% absolute (24% relative) increase in a national emergency department dataset.

**Conclusions:**

The updated CCC v2 system is comprehensive and multidimensional, and provides a necessary update to accommodate widespread implementation of ICD-10.

## Background

Remarkable advances in pediatric healthcare over the past century have substantially reduced overall childhood morbidity and mortality, while concomitantly enabling some children with complex chronic conditions (CCC) to survive and live longer. A clear definition of these CCCs is important for several reasons: to assess population-level temporal trends in the proportion of morbidity and mortality associated with CCCs; to assess patterns of healthcare utilization among patients with CCCs; and to perform individual-level risk adjustments for patients’ CCC status in studies of healthcare processes and outcomes.

In 2000, Feudtner and colleagues developed a definition for CCCs: “Any medical condition that can be reasonably expected to last at least 12 months (unless death intervenes) and to involve either several different organ systems or 1 organ system severely enough to require specialty pediatric care and probably some period of hospitalization in a tertiary care center”[1]. Based on this definition, a comprehensive set of codes available at that time from the International Classification of Disease version 9 Clinical Modification (ICD-9-CM) system were identified as indicative of a CCC, and further categorized into 9 categories (cardiovascular, respiratory, neuromuscular, renal, gastrointestinal, hematologic or immunologic, metabolic, other congenital or genetic, and malignancy).

The CCC classification system (v1) can be used to either examine a particular CCC category or to identify patients with multiple CCC categories. The CCC system was initially applied to studying patterns of pediatric mortality and end-of-life care [1,2], and has subsequently been applied to a variety of research problems, including risk adjustment, prediction of adverse health outcomes, and identification of populations with high health care utilization [3-27].

The original CCC system, now more than a decade old, warranted revision for 3 main reasons. First, the International Classification of Disease 10th Revision (ICD-10) is replacing the ICD-9 system in the United States. Although already widely used internationally, ICD-10 was first used in the Centers for Disease Control and Prevention vital statistics data files in 1999, and government diagnostic and procedural billing codes will change to ICD-10 in 2015. Second, the original CCC system has not been updated to include new diagnostic codes added over the past decade, and in particular the original CCC system does not include a category for conditions originating in the neonatal period. Third, the original CCC system consisted entirely of diagnostic codes and lacked a domain of codes that specify either a likely dependence upon medical technology or having undergone organ (including bone marrow) transplantation, both of which would indicate CCC status.

In this study, we report on our updated CCC version 2 (v2) classification system, which we implemented for both the ICD-9 and ICD-10 coding schemes. We evaluated the CCC v2 system in three ways: 1) the comparability between the ICD-9 and ICD-10 systems; 2) the year-to-year stability of classification across CCC v2 attributable to cause of death in 1991–2010; and 3) the proportions of patients classified as having a CCC between CCC v2 and CCC v1 systems.

## Methods

### Institutional review board

This research was exempt by the Children’s Hospital of Philadelphia Institutional Review Board.

### Development of CCC V2 classification scheme

We updated the CCC classification system in 5 steps. First, we reviewed publications that either used or evaluated the CCC v1 system, identifying ICD-9 codes that have been found to be omitted in the CCC v1 system but likely to indicate CCC status, and incorporated these codes. Second, we translated ICD-9 to ICD-10 codes by using GEMs (General Equivalence Mappings) forward mapping. The GEMs for the diagnosis codes are developed by the National Center for Health Statistics (NCHS); the GEMs for the procedure codes are developed by the Centers for Medicare & Medicaid Services (CMS). They are the authoritative source for translation between ICD-9 and ICD-10 codes (http://www.cdc.gov/nchs/icd/icd10cm.htm.). Third, two member of the team (CF and JF), both experienced clinicians who care exclusively for children with CCCs, independently reviewed all codes in the ICD-9 and ICD-10 taxonomies to identify neonatal conditions, specifically those arising in the perinatal period (including very low birth weight) that have a high likelihood of meeting the CCC definition. All discrepancies were resolved through further query of the detailed ICD taxonomies (http://www.who.int/classifications/icd/en/). Fourth, in a similar manner but this time focusing on technology dependence and transplantation status, the same team members (CF and JF) reviewed ICD-9 and ICD-10 codes, as well as existing publications [10,28,29], to identify codes indicative of persons who likely have a CCC. Fifth, with the provisional set of all CCC v2 codes, we used the v2 system to classify all cases in the CDC Multiple Cause of Death data for 1996 (http://wonder.cdc.gov/mortSQL.html), 2009 Kids’ Inpatient Database (KID), and 2010 Nationwide Emergency Department Sample (NEDS) datasets (http://www.hcup-us.ahrq.gov/databases.jsp), and reviewed all cases classified as *not* having a CCC to determine whether any codes in the CCC v2 system had been either incorrectly specified or omitted, and then we corrected these errors.

### Evaluation of V2 classification scheme

We evaluated the CCC v2 system in three important ways on diverse data sources. First, we evaluated the CCC v2 system in terms of comparability between ICD-9 and ICD-10 codes using the CDC Multiple Cause of Death data for 1996, which is coded using both ICD-9 and ICD-10 schemes (ftp://ftp.cdc.gov/pub/health_statistics/nchs/datasets/Comparability/icd9_icd10; more about the comparability ratio can be referred to: http://www.ons.gov.uk/ons/guide-method/classifications/international-standard-classifications/icd-10-for-mortality/comparability-ratios/index.html). We estimated the comparability ratio to determine whether any discrepancies between ICD-9 and ICD-10 were of significance (Z-test) [30]. The comparability refers to the ratio of the number of a CCC coded from ICD-9 to the number of the equivalent CCC coded from ICD-10. For any CCC category (CR_c_), the comparability ratio is calculated as CR_*c*_ = D_c_^ICD-10^/D_c_^ICD-9^, where D_c_^ICD-10^ is the number of deaths attributable to CCC v2 classified by ICD-10 and D_c_^ICD-9^ is the number of deaths attributable to CCC v2 classified by ICD-9. A comparability ratio of 1.00 indicates that the same number of deaths was assigned to the CCC category under both ICD-9 and ICD-10, denoting no net effect of ICD-10 on that particular CCC category. A comparability ratio less than 1.00 results from fewer deaths being classified to that CCC category under ICD-10, and a ratio greater than 1.00 indicates more deaths being classified under ICD-10.

Second, we examined the year-to-year stability of classification across the 1999 implementation of ICD-10 for the CDC Multiple Cause of Death data from 1991–2010. In this data source, coding of mortality causes transitioned from ICD-9 to ICD-10 in 1999. Therefore, we identified the CCC v2 categories with ICD-9 before 1999, and with ICD-10 beginning in 1999. The proportions of the ten CCC v2 categories for each year were estimated, and were then plotted against the year (1991–2010). We examined changes in the time trends of CCC v2 proportions, particularly at the 1999 time point.

Finally, we compared the proportions of CCC v2 categories to those of CCC v1 using the KID 2009 and the NEDS 2010 data. CCC categories were identified using the v1 and v2 systems based on ICD-9 codes, since these datasets do not yet contain ICD-10 codes. We assessed for differences between the resulting estimates and reported the absolute and relative change in percentages between the two systems for each of the datasets.

All database management and data analysis were conducted using SAS 9.3 (SAS Institute Inc, Cary, North Carolina) \and Stata 12.1 (StataCorp, College Station, Texas). The SAS macro (ccc_version2_sas) and Stata do file (ccc_version2_stata) for CCC v2 are available as supplemental files (see Additional files 1, 2, 3 and 4).

## Results

### Updated CCC classification system: CCC version 2 (CCC v2)

The CCC v2 classification system consists of ICD-10 codes as well as ICD-9 codes (see Additional file 5). CCC v2 has ten CCC categories: the original 9 (cardiovascular, respiratory, neuromuscular, renal, gastrointestinal, hematologic or immunologic, metabolic, other congenital or genetic, and malignancy) and a new category of conditions (premature and neonatal) arising in the perinatal period that are indicative of likely CCC status. Several additional diagnoses indicative of a CCC were added in CCC v2 to specific CCC categories. Under each CCC category, subcategories are provided for more refined grouping. In addition, CCC v2 also includes a domain of complexity arising from dependence upon medical technology (device), and a domain indicating having received transplantation. The codes indicating these states are included as subcategories under each CCC category, and also as a separate group of “Miscellaneous, Not Elsewhere Classified”, which refers to device/technology dependence and transplantation.

### Comparability of CCC v2 between ICD-9 and ICD-10

When applied to the CDC Multiple Cause of Death for 1996, the ICD-9 and ICD-10 versions of CCC v2 identified similar numbers of affected patients (Table 1). In some CCC categories (such as neurologic CCC), ICD-10 identified slightly fewer patients, while in some categories (such as premature and neonatal CCC), ICD-10 identified slightly more patients; none of these differences were statistically significant. The comparability ratios ranged between 0.95 and 1.05.

**Table 1:** Estimated comparability ratios for 10 categories of CCC v2 classified by underlying cause of death ICD-9 and ICD-10 codes in the CDC Multiple Cause of Death data for 1996

	**Number allocated (N = 56,099)**			
**CCC categories**	**ICD-9**	**ICD-10**	**Comparability ratio (95% CI)**	***p *** **value**
Neurologic and neuromuscular	2,466	2,337	0.9477 (0.8967–1.006)	0.0571
Cardiovascular	3,423	3,397	0.9924 (0.9478–1.0391)	0.7453
Respiratory	1,447	1,378	0.9523 (0.8854–1.0243)	0.1886
Renal and urologic	356	369	1.0364 (0.8965–1.1984)	0.6281
Gastrointestinal	173	177	1.0231 (0.8300–1.2612)	0.8304
Hematologic or immunologic	691	724	1.0476 (0.9447–1.1621)	0.774
Metabolic	274	273	0.9963 (0.8429–1.1777)	1.000
Other congenital or Genetic defect	2,095	2,061	0.9838 (0.9268–1.0443)	0.591
Malignancy	2,443	2,549	1.0437 (0.9883–1.1016)	0.1249
Premature and neonatal	949	995	1.0485 (0.9600–1.1451)	0.2926
Technology dependence	6	6	1.0000 (0.3225–3.1004)	1.000
Transplantation	0	0	---	---
CCC (Any CCC)	14,674	14,639	0.9976 (0.9782–1.0174)	0.8120

The comparability ratio for any CCC category (CR_c_) is calculated as CR_*c*_ = D_c_^ICD-10^/D_c_^ICD-9^, where D_c_^ICD-10^ is the number of deaths attributable to CCC category classified by ICD-10 and D_c_^ICD-9^ is the number of deaths attributable to CCC category classified by ICD-9.

### Temporal trends of CCC v2 attributable to cause of death in 1991–2010

**Figure 1:**
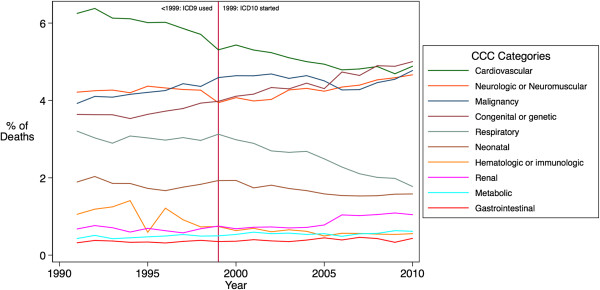
Trends of CCC v2 categories in CDC Multiple Cause of Death data 1991–2010.

From the graphic presentation, we did not observe substantial increases or decreases in the year-to-year stability of CCC v2 classifications across the year 1999 when ICD-10 was implemented, viewed against the overall year-to-year pattern of change of CCC v2 proportions during this 20-year period (Figure 1). Overall, the proportion of death attributed to each CCC v2 category remained fairly stable across the years, with minor decline in cardiovascular CCC and respiratory CCC, and a slight increase in congenital and genetic CCC. The proportion of the overall population with one or more CCC remained stable, and ranged from 24.4% to 26.1% during 1991–2010.

### Proportions of CCC categories between CCC v2 and CCC v1

In general, CCC v2 classified more patients as having complex chronic conditions than CCC v1 (except for the malignancy and cardiovascular CCC categories) (Table 2). In absolute terms, CCC v2 identified 1.0% more patients as having a CCC in the KID 2009, and a 0.4% in the NEDS 2010, compared to CCC v1. In relative terms, 10% more patients in the KID 2009 and 24% in the NEDS 2010 were identified as having a CCC in CCC v2 than in CCC v1. The largest increase was in the gastrointestinal CCC, with a 226% increase in the KID, and a 333% increase in the NEDS. The second largest increase was the neurologic and neuromuscular CCC, with a 68% increase in both the KID and the NEDS. In contrast, congenital CCC had no change between CCC v1 and v2 in both the KID and the NEDS.

**Table 2:** Percentages of patients with CCCs classified by CCC v1 versus CCC v2 in KID 2009 and NEDS 2010

**CCC categories**	KID* (Weighted N = 7,337,017)			**NEDS (Weighted N = 29,741,986)**		
	**CCC v1**	**CCC v2**	**% Change**	**CCC v1**	**CCC v2**	**% change**
Neurologic and neuromuscular	1.36 (1.35–1.37)	2.28 (2.26–2.30)	67.64%	0.31 (0.26–0.37)	0.52 (0.43–0.61)	67.74%
Cardiovascular	3.29 (2.27–3.30)	2.32 (2.30–2.33)	–29.48%	0.43 (0.38–0.48)	0.48 (0.42–0.54)	11.63%
Respiratory	0.92 (0.91–0.93)	0.97 (0.96–0.98)	5.43%	0.08 (0.07–0.11)	0.13 (0.10–0.16)	62.50%
Renal and urologic	0.75 (0.74–0.76)	1.21 (1.12–1.22)	61.33%	0.08 (0.07–0.10)	0.13 (0.11–0.15)	62.50%
Gastrointestinal	0.62 (0.61–0.63)	2.02 (2.00–2.03)	225.81%	0.09 (0.08–0.10)	0.39 (0.30–0.48)	333.33%
Hematologic or immunologic	1.32 (1.30–1.33)	1.60 (1.58–1.61)	21.21%	0.34 (0.28–0.39)	0.41 (0.35–0.47)	20.59%
Metabolic	0.84 (0.83–0.85)	1.11 (1.10–1.12)	32.14	0.14 (0.12–0.16)	0.19 (0.16–0.21)	35.71%
Other congenital or genetic defect	1.64 (1.63–1.65)	1.64 (1.63–1.65)	0.00%	0.31 (0.27–0.36)	0.31 (0.27–0.36)	0.00%
Malignancy	2.10 (2.08–2.11)	1.64 (1.63–1.65)	–21.91%	0.21 (0.17–0.25)	0.16 (0.12–0.20)	-23.81%
Neonatal	---	1.14 (1.13–1.15)	---	---	0.03 (0.02–0.04)	---
Technology dependency	---	2.64 (2.62–2.65)	---	---	0.46 (0.35–0.58)	---
Transplantation	---	0.38 (0.37–0.38)	---	---	0.05 (0.04–0.07)	---
CCC (Any CCC)	10.56 (10.53–10.59)	11.57 (11.53–11.59)	9.56%	1.83 (1.60–2.06)	2.27 (1.97–2.72)	24.04%

Legend: Data presented as percentage (% (95% CI) ). CCCv2 is classified using ICD-9. * Included all KID patients.

## Discussion

Children with complex chronic conditions present challenges and opportunities in delivering healthcare, which necessitates classification schemes that can distinguish children across a spectrum of pediatric health states [31,32]. This spectrum of health states comprises several related but distinct dimensions: the degree of functional capacity or impairment across several domains of function; the hazard that a disease or condition poses to function or life; and the ease or difficulty of curing or ameliorating the disease. These dimensions are bundled into the notion of *health state* and the related concept of a *health (or illness) trajectory* (a phrase that we are using to connote the likely pattern of change of health over time).

The multidimensional nature of both health states and health trajectories reinforces that no single uniform classification system is likely to serve all the different goals of research or quality improvement projects that study the needs of children with complex chronic conditions. Instead, an ideal system should be flexible and capable of being adapted to address each particular research question. The system should also be transparent regarding how it is constructed, implemented, and subsequently audited, which can be facilitated by providing open source code that can be reviewed and modified by end-users. Finally, the system should be multidimensional, meaning that the system should capture different components of health states and health trajectories.

The original CCC v1 system aimed to identify infants, children, and adolescents diagnosed with complex chronic conditions, with an emphasis on examining patterns of mortality and of end-of-life healthcare utilization associated with CCCs. The system was built around ICD-9 codes, which were grouped into categories of disease to track different anatomical and physiologic pathologies associated with the different health states and trajectories.

The CCC system is flexible in that this classification system can be used to examine a particular CCC category or to identify patients with multiple CCC categories and significant multisystem comorbidities. Patients with >1 CCC category have a higher risk of increased inpatient readmission rate and ultimately death [22,33,34]. Furthermore, pediatric patients with multiple CCC categories are increasingly prevalent in US hospitals [35]. For datasets where individuals can be tracked over multiple encounters, the CCC system also can be used to classify patients based on their cumulative CCC status aggregated over time.

The updated CCC v2 is more comprehensive than the original CCC system, having incorporated additional new CCC diagnoses, a category of neonatal CCCs, and domains for technology dependent or post-transplant related conditions. The addition of the neonatal category caused several CCC diagnoses to shift to this new category (for example, chronic respiratory disease arising in the perinatal period [ICD-9: 770.7] had been classified as a “respiratory” CCC in CCC v1, but now is classified as a “neonatal” CCC in CCC v2). In future studies with CCC v2, however, investigators should keep in mind that the increased sensitivity in identifying children with complex chronic conditions may have decreased specificity.

The domain of technology-dependent and post-transplant related condition captures conditions that are increasingly prevalent with advances in medical technology and treatments [29]. A single institution study reported that 41% of patients discharged from a children’s hospital in 2000 were dependent upon medical technology (20% upon medical devices, 32% upon medicines, and 11% upon both devices and medicines) [28]. A national study revealed that in 2006, patients with CCCs accounted for 73% of gastrostomy tube placements, 75% of tracheostomy tube placements, and 92% of cerebrospinal fluid shunts [22]. By identifying this population of CCC patients, we will be able to better evaluate the experiences of technology-dependent and transplanted children, examine their patterns of health care utilization, and design programs to provide coordinated, efficient, and cost-effective care.

Importantly, the CCC v2 system can be implemented for either the ICD-9 or the ICD-10 coding scheme. With the mandated transition from ICD-9 to ICD-10 coding schemes for medical diagnoses and procedures, the CCC v2 system provides a necessary update. We tested the comparability of CCC v2 between ICD-9 and ICD-10, and found no significant discrepancies in CCC categories between the ICD-9 and ICD-10 schemes. We also examined the temporal trends of CCC categories attributable to cause of death during 1991–2010, and did not find any drastic changes at the 1999 time point, when ICD-10 coding was implemented, and no discontinuities in long-term time trends.

We did observe minor declines in the cardiovascular and respiratory CCC categories in the CDC Multiple Cause of Death 1991–2010, which corresponded with a reduction in overall population death rates in heart disease and chronic lower respiratory diseases [36], which have been attributed in part to reductions in tobacco use [37,38], but for pediatric patients, are more likely due to improved cardiac and pulmonary care. The minor increases in the congenital/genetic CCC were most likely related to improved diagnostic capabilities, such as the improved detection of genetic diseases through advancements in newborn screening processes. Nevertheless, none of these trends demonstrated substantial shifts over the 20-year period. This suggests that CCC v2 has close comparability between ICD-9 and ICD-10, and that the transition from ICD-9 to ICD-10 is unlikely to substantially change CCC v2 proportions, or cause a significant discontinuity in time trends.

We also measured the degree to which the CCC v2 system identified more patients with complex chronic conditions than CCC v1 because of the modifications and additions. While most CCC categories demonstrated moderate or almost no change (such as the congenital/genetic CCC) in the percentage of all deaths attributed to a given CCC category, there were several CCC categories had notable increases in percentages when the CCC v2 system was applied (such as the gastrointestinal CCC); and the change in the percentage of cases assigned to a CCC category also varied when the schemes were applied to different national data sources (for example, the percentage of patients with gastrointestinal CCC increased 226% in the KID but increased 333% in the NEDS). Cardiovascular CCC and malignancy CCC both decreased in NEDS and KID. These decreases were due to the elimination of self-limiting or completely correctable cardiovascular conditions (ICD-9: 745.4, 745.5, 747.0, and 745.7) and benign neoplasms (ICD-9: 210–229) in the CCC v2 scheme.

## Conclusions

The CCC v2 system is more comprehensive and provides a necessary update for the government-mandated transition from ICD-9 to ICD-10. The CCC v2 system showed close comparability between ICD-9 and ICD-10, and did not cause substantial discontinuity in the temporal trends of CCC prevalence. We hope that this updated CCC classification system will be sufficiently transparent and flexible to serve the next generation of research about children with complex chronic conditions.

## Abbreviations

CCC: Complex chronic condition; CMS: Centers for Medicare & Medicaid Services; CR: Comparability ratio; GEM: General equivalence mappings; ICD-9: International Classification of Disease 9th Revision; ICD-10: International Classification of Disease 10th Revision; KID: Kids’ Inpatient Database; NCHS: National Center for Health Statistics; NEDS: Nationwide Emergency Department Sample.

## Competing interests

The authors declare that they have no relevant financial relationships and no relevant non-financial relationships to disclose.

## Authors’ contributions

All authors contributed to the conception and design of the study, implemented different aspects of the analysis, drafted sections of the manuscript, revised the manuscript, and approved the final version for submission.

## Pre-publication history

The pre-publication history for this paper can be accessed here:

http://www.biomedcentral.com/1471-2431/14/199/prepub

## Supplementary Material

Additional file 1 **CCC V2 SAS Program.** Actual SAS macro program code to apply the CCC V2 system to user data. Click here for file

Additional file 2 **CCC V2 SAS Program.** PDF SAS macro program code to apply the CCC V2 system to user data. Click here for file

Additional file 3 **CCC V2 Stata Program.** Actual Stata macro program code to apply the CCC V2 system to user data. Click here for file

Additional file 4 **CCC V2 Stata Program.** PDF Stata macro program code to apply the CCC V2 system to user data. Click here for file

Additional file 5 **Categories of CCC v2 and the Corresponding ICD-9 and ICD-10 Diagnosis and Procedure Codes.** Table of CCC categories and the corresponding ICD-9 and ICD-10 diagnosis and procedure codes. Click here for file
