# Supplementary material for: Pediatric complex chronic conditions classification system version 2: updated for ICD-10 and complex medical technology dependence and transplantation
Source: BMC Pediatr. 2014 Aug 8;14:199. doi: 10.1186/1471-2431-14-199 (PMC4134331; doi:10.1186/1471-2431-14-199)
Supplement: Additional file 2 — CCC V2 SAS Program. PDF SAS macro program code to apply the CCC V2 system to user data. [file 1471-2431-14-199-S2.pdf]

```
/
**********************************************************************
*****************************
Macro Name: ccc_version2_sas

Function:
This program will be used to create CCC and CCC subcategories flag and
compute number of ccc
category based on articles:
Feudtner C, et al. Pediatric complex chronic conditions classification
system version 2:
updated for ICD-10 and complex medical technology dependence and
transplantation.

Author: Dingwei Dai

Date: 2014-04-09

Call statement:
%ccc_version2_sas(dt_in,dt_out,dx,n_dxs,pc,n_pcs,icdv)
run;
Parameter definitions:
   dt_in  SAS input data set containing patient id and all ICD-9-CM or
ICD-10-CM codes
   dt_out SAS output data set containing all input data and new
created CCC and CCC subcategories
          flags and number of subcategory
   dx prefix for ICD-9-CM or ICD-10-CM diagnosis code
   n_dxs number of ICD-9-CM or ICD-10-CM diagnosis code (assume
diagnosis variables as dx1 - dxn)
   pc prefix for ICD-9-CM or ICD-10-CM procedure code
   n_pcs number of ICD-9-CM or ICD-10-CM procedure code (assume
procedure variables as px1 - pxn)
   icdv ICD version 9 or 10

For example, if you use KID2009, just call:
%ccc_version2_sas(kid_2009_core,kid_2009_core_results,dx,25,pr,15,9)
**********************************************************************
*****************************/

%macro ccc_version2_sas(dt_in,dt_out,dx,n_dxs,pc,n_pcs,icdv);

data &dt_out;
   set &dt_in;

   neuromusc_ccc=0;
   cvd_ccc=0;
   respiratory_ccc=0;
   renal_ccc=0;
   GI_ccc=0;
```

```
   hemato_immu_ccc=0;
   metabolic_ccc=0;
   congeni_genetic_ccc=0;
   malignancy_ccc=0;
   neonatal_ccc=0;
   tech_dep_ccc=0;
   transplant_ccc=0;

   *** diagnosis codes;
   array dxc(&n_dxs.) &dx.1 - &dx.&n_dxs.;
   *** procedure codes;
   array pcc(&n_pcs.) &pc.1 - &pc.&n_pcs.;

   if &icdv=9 then do;

   do i=1 to &n_dxs.;

   if dxc(i) in:
("3180","3181","3182","330","33111","33119","3314","33189","3319","332
0","3321",

"3330","3332","3334","3335","3337","3339","334","335","343","34501","3
4581","3590","3591",

"3592","3593","3361","3368","3379","3418","34290","343","3440","34481"
,"3449","34511",

"3453","34541","34561","34571","34591","3481","3484","3491","43401","4
3491","359","740",

"741","742","7595","78003","9962","99663","V452","V5301","V5302") then
neuromusc_ccc=1;

   else if dxc(i) in:
("4161","4168","4169","4240","4258","4242","4243","4250","4251","4252"
,"4253",

"4254","426","4270","4271","4272","4273","4274","4276","4277","4278","
4279","4280","42883",

"4291","4293","43311","7450","7451","7452","7453","7456","746","7471",
"7472","7473","7474",

"74781","74789","9960","9961","99661","99662","V421","V422","V432","V4
33","V450","V4581",
          "V533") then cvd_ccc=1;

   else if dxc(i) in:
("32725","4162","5163","51631","51884","5190","2770","748","7704","V42
6",
```

```
         "V440","V4576","V460","V461","V550") then respiratory_ccc=1;

   else if dxc(i) in:
("34461","585","5964","59653","59654","753","99668","V420","V445","V44
6",
         "V451","V4573","V4574","V536","V555","V556","V56") then
renal_ccc=1;

   else if dxc(i) in:
("4530","5364","555","556","5571","5602","5647","5714","5715","5716","
5717",

"5718","5719","7503","751","V427","V4283","V4284","V441","V442","V443"
,"V444","V5350",
         "V5351","V5359","V551","V552","V553","V554") then GI_ccc=1;

   else if dxc(i) in:
("042","043","044","135","279","2820","2821","2822","2823","2824","282
5",

"2826","2881","2882","284","2860","2863","28732","28733","28739","2880
1","28802",

"2884","4460","4461","44621","4464","4465","4466","4467","7100","7101"
,"7103","V08")
         then hemato_immu_ccc=1;

   else if dxc(i) in:
("243","2532","2535","2536","2359","2550","25513","2552","270","271","
272",

"2750","2751","2752","2753","2772","2773","2774","2775","2776","2778",
"2779",
         "V4585","V5391","V6546") then metabolic_ccc=1;

   else if dxc(i) in:
("2594","5533","7373","7560","7561","7562","7563","7564","7565","7566"
,
         "7567","758","7597","7598","7599") then
congeni_genetic_ccc=1;

   else if dxc(i) in:
("14","15","16","17","18","19","20","23","V4281","V4282")
         then malignancy_ccc=1;

   else if dxc(i) in:
("76401","76402","76411","76412","76421","76422","76491","76492","7650
1",

"76502","76511","76512","76521","76522","76523","7670","7674","7685","
```

```
7687","7689",

"7702","7707","7710","7711","77213","77214","7733","7734","7747","7765
","77753","7780",
          "7797") then neonatal_ccc=1;

   if dxc(i) in:
("5190","5364","9960","9961","9962","9964","99661","99662","99663","99
666","99667",

"99668","9969","V433","V440","V441","V442","V443","V444","V445","V446"
,"V450","V451",

"V452","V460","V461","V462","V5301","V5302","V5331","V5332","V5339","V
5350","V5351",

"V5359","V536","V550","V551","V552","V553","V554","V555","V556") then
tech_dep_ccc=1;

   if dxc(i) in:
("99680","99681","99682","99683","99684","99685","99686","99687","9968
8","99689",

"V421","V422","V426","V427","V4281","V4282","V4283","V4284","V432","V4
585","V5391") then transplant_ccc=1;

   end;

   do i=1 to &n_pcs.;

   if pcc(i) in:
("0152","0153","0221","0222","0231","0232","0233","0234","0235","0239"
,"0241",
          "0242","0293","0371","0372","0379","0393","0397","0492")
then neuromusc_ccc=1;

   else if pcc(i) in:
("0050","0051","0053","0054","0055","0057","1751","1752","3581","3582"
,"3583",

"3584","3741","3751","3752","3753","3754","3755","3760","3761","3763",
"3765","3766",

"3767","3768","3771","3772","3774","3776","3779","3780","3781","3782",
"3783","3785",

"3786","3787","3789","3794","3795","3796","3797","3798","3981","3982",
"3983","3984",
          "3985","8945","8946","8947","8948","8949") then cvd_ccc=1;
```

```
   else if pcc(i) in:
("303","304","3121","3129","3141","3174","3241","3249","3250","3259","
3321",
          "3350","3351","3352","336", "3485","9655","9723") then
respiratory_ccc=1;

   else if pcc(i) in:
("3895","3927","3942","3993","3994","3995","5498","5502","5503","5504"
,"5512",

"5551","5552","5553","5554","5561","5569","5593","5594","5597","5641",
"5642","5651","5652",

"5661","5662","5671","5672","5673","5674","5675","5679","5721","5722",
"5771","5779","5993",
          "5994","8607","9645","9646","9647") then renal_ccc=1;

   else if pcc(i) in:
("253","254","4210","4211","4242","4281","4311","4319","4391","4399","
4412",

"4432","4438","4439","4563","4581","4582","4583","4613","4622","4623",
"4632","4640","4641",
          "4643","4697","504",
"5051","5059","526","527","5280","5282","5283","5284","5285","5286",
          "5471","9624","9636","9702") then GI_ccc=1;

   else if pcc(i) in:
("4100","4101","4102","4103","4104","4105","4106","4107","4108","4109"
,"415",
          "4194") then hemato_immu_ccc=1;

   else if pcc(i) in:
("064","0652","0681","073","0764","0765","0768","0769","624","6241","6
45","6551",

"6553","6561","6563","6841","6849","6851","6859","6861","6869","6871",
"6879","8606") then metabolic_ccc=1;

   else if pcc(i) in: ("0010","9925") then malignancy_ccc=1;

   if pcc(i) in
("0221","0222","0231","0232","0233","0234","0235","0239","0241","0242"
,"0293","0371",

"0372","0379","0393","0397","0492","0050","0051","0053","0054","0055",
"0057","1751","1752",

"3741","3752","3753","3754","3755","3760","3761","3763","3765","3766",
"3767","3768","3771",
```

```
"3772","3774","3776","3779","3780","3781","3782","3783","3785","3786",
"3787","3789","3794",

"3795","3796","3797","3798","3981","3982","3983","3984","3985","8945",
"8946","8947","8948",

"8949","3121","3129","3141","3174","3321","3485","9655","9723","3895",
"3927","3942","3993",

"3994","3995","5498","5502","5503","5504","5512","5593","5594","5597",
"5651","5652","5661",

"5662","5672","5673","5674","5675","5721","5722","5993","5994","8607",
"9645","9646","9647",

"4210","4211","4281","4311","4319","4412","4432","4438","4439","4613",
"4622","4623","4632",

"4640","4641","4643","9624","9636","9702","8100","8101","8102","8103",
"8104","8105","8106",

"8107","8108","8109","8130","8131","8132","8133","8134","8135","8136",
"8137","8138","8139",
          "8451") then tech_dep_ccc=1;

   if pcc(i) in ("3751","3350","3351","3352","336",
"5561","5569","4697","5051","5059","5280","5282",

"5283","5284","5285","5286","4100","4101","4102","4103","4104","4105",
"4106","4107","4108",
          "4109","4194","0091","0092","0093") then transplant_ccc=1;

   end;

   end;

   else if &icdv=10 then do;

   do i=1 to &n_dxs.;

   if dxc(i) in:
("E750","E751","E752","E754","F71","F72","F73","F842","G111","G112","G
114","G118",

"G119","G120","G121","G122","G128","G129","G3101","G3109","G318","G318
9","G3289","G71",

"G72","G80","G901","G938","G939","G94","Q00","Q01","Q02","Q03","Q04","
Q05","Q06","Q07",
```

```
"G911","G319","G253","G9519","G9589","G909","G40311","G40301","G40211"
,"G40219","G40411",

"G40419","G40804","G40111","G40119","G40911","G40919","G371","G372","G
378","G8190","G8290",

"G8250","G8251","G8252","G8253","G8254","G835","G839","G931","G935","I
6330","I6350","G10",

"G20","G210","G2111","G2119","G218","G230","G231","G232","G238","G2402
","G248","G253",

"G254","G255","G2581","G2582","G2583","G2589","G259","G803","R403","G9
782","T8509XA",

"T85190A","T85192A","T85199A","T8579XA","Z982","Z4541","Z4542") then
neuromusc_ccc=1;

   else if dxc(i) in:
("I270","I271","I272","I2781","I2789","I279","I340","I348","I360","I36
8","I370",

"I378","I42","I43","I44","I45","I47","I48","I490","I491","I493","I494"
,"I495","I498","I499",

"I509","I515","I517","I5181","I63139","I63239","Q20","Q212","Q213","Q2
14","Q218","Q22","Q23",

"Q24","Q251","Q252","Q253","Q254","Q255","Q256","Q257","Q26","R001","Q
282","Q283","Q289",

"Z951","T82519A","T82529A","T82539A","T82599A","T82110A","T82111A","T8
2120A","T82121A",

"T82190A","T82191A","T8201XA","T8202XA","T8203XA","T8209XA","T82211A",
"T82212A","T82213A",

"T82218A","T82221A","T82222A","T82223A","T82228A","T82518A","T82528A",
"T82538A","T82598A",

"T826XXA","T827XXA","Z941","Z950","Z952","Z95810","Z95811","Z95812","Z
95818","Z953","Z45010",
          "Z45018","Z4502","Z4509","Z959") then cvd_ccc=1;

   else if dxc(i) in:
("E84","G4753","I2782","I43","Q30","Q31","Q32","Q33","Q34","P280","Z90
2","J84112",

"J9500","J9501","J9502","J9503","J9504","J9509","9620","Z430","Z930","
```

```
Z942","Z990","J95850",
          "Z9911","Z9912","T86810","T86811","T86819") then
respiratory_ccc=1;

   else if dxc(i) in:
("N18","Q60","Q61","Q62","Q63","Q64","Z905","Z906","G834","N312","N319
","T8571XA",

"Z940","Z9350","Z9351","Z9352","Z9359","Z936","Z9115","Z992","Z435","Z
436","Z446","Z4901",
          "Z4902","Z4931","Z4932","T8610","T8611","T8612") then
renal_ccc=1;

   else if dxc(i) in:
("K50","K51","K73","K74","K754","K760","K761","K762","K763","K768","Q3
90","Q391",

"Q392","Q393","Q394","Q41","Q42","Q43","Q44","Q45","I820","K551","K562
","K593","Z980","Z903",

"Z9049","K9420","K9422","K9423","K9429","Z944","Z9482","Z9483","Z931",
"Z932","Z933","Z934",

"Z431","Z432","Z433","Z434","Z4651","Z4659","T8640","T8641","T8642","T
86890","T86891",
          "T86899","T86850","T86851","T86859") then GI_ccc=1;

   else if dxc(i) in: ("B20",
"D55","D56","D57","D58","D60","D61","D71","D720","D80","D81","D82",

"D83","D84","D85","D87","D88","D86","M303","M359","B21","B22","B23","B
24","D700","D704",
          "D66",
"D682","D6941","D6942","D761","D762","D763","D869","M300","M310","M311
","M3130","M314",
          "M316","M3210","M3390","M340","M341","M349","Z21") then
hemato_immu_ccc=1;

   else if dxc(i) in:
("D841","E700","E702","E703","E704","E705","E708","E710","E711","E712"
,"E713",

"E714","E715","E720","E721","E722","E723","E724","E728","E729","E740",
"E741","E742","E743",

"E744","E748","E749","E75","E760","E761","E762","E763","E770","E771","
E780","E781","E782",

"E783","E784","E785","E786","E787","E788","E789","E791","E798","E804",
"E805","E806","E807",
```

```
"E830","E831","E833","E834","E88","H498","E85","E009","E230","E232","E
222","E233","E237",

"E240","E242","E243","E248","E249","E2681","E250","E258","E259","Z4681
","Z794","Z9641") then metabolic_ccc=1;

   else if dxc(i) in:
("E343","K449","M410","M412","M4130","M418","M419","M4330","M965","Q72
2","Q750",

"Q752","Q759","Q760","Q761","Q762","Q764","Q765","Q766","Q767","Q77","
Q780","Q781","Q782",

"Q783","Q784","Q788","Q789","Q790","Q791","Q792","Q793","Q794","Q799",
"Q795","Q8740","Q8781",

"Q8789","Q897","Q899","Q909","Q913","Q914","Q917","Q928","Q93","Q950",
"Q969","Q97","Q98",
         "Q992","Q998","Q999","Q898","Q81") then
congeni_genetic_ccc=1;

   else if dxc(i) in:
("C","D00","D01","D02","D03","D04","D05","D06","D07","D08","D09","D37"
,"D38",

"D39","D3A0","D40","D41","D42","D43","D44","D45","D46","D47","D48","D4
9","Q850","Z9481",
         "Z9484","T8600","T8601","T8602","T8609") then
malignancy_ccc=1;

   else if dxc(i) in:
("P0501","P0511","P0502","P0512","P052","P059","P0701","P0702","P0721"
,"P0722",

"P0723","P0724","P0725","P100","P101","P104","P524","P528","P115","P84
","P916","P210","P250",

"P251","P253","P258","P219","P270","P271","P278","P350","P351","P2521"
,"P2522","P560","P570",
         "P578","P613","P614","P773","P832","P912") then
neonatal_ccc=1;

   if dxc(i) in:
("T8509XA","T85190A","T85192A","T85199A","T8579XA","Z982","Z45441","Z4
5442","T82519A",

"T82529A","T82539A","T82599A","T82110A","T82111A","T82120A","T82121A",
"T82190A","T82191A",
```

```
"T8201XA","T8202XA","T8203XA","T8209XA","T82211A","T82212A","T82213A",
"T82218A","T82221A",

"T82222A","T82223A","T82228A","T82518A","T82528A","T82538A","T82598A",
"T826XXA","T827XXA",

"Z950","Z952","Z953","Z95810","Z95811","Z95812","Z95818","Z45010","Z45
018","Z4502","Z4509",

"Z959","J9500","J9501","J9502","J9503","J9504","J9509","Z430","Z930","
Z942","Z990","J95850",

"Z9911","Z9912","T8571XA",'Z940',"Z9350","Z9351","Z9352","Z9359","Z936
","Z9115","Z992",

"Z435","Z436","Z446","K9420","K9422","K9423","K9429","Z931","Z932","Z9
33","Z934","Z431",

"Z432","Z433","Z434","Z4651","Z4659","Z4681","Z9641","T84019A","T84029
A","T84039A","T84049A",

"T84059A","T84069A","T84099A","T84498A","T84119A","T84129A","T84199A",
"T84498A","T8450XA",

"T8460XA","T847XXA","T8690","T8691","T8692","T8699","T8610","T8611","T
8612","T8640","T8641",

"T8642","T8620","T8621","T8622","T86810","T86811","T86819","T8600","T8
601","T8602","T8609",

"T86890","T86891","T86899","T86850","T86851","T86859","T865","T86890",
"T86891","T86899",
          "T870X9","T871X9","T872","Y831","Y833") then tech_dep_ccc=1;

   if dxc(i) in:
("T8600","T8601","T8602","T8609","T8610","T8611","T8612","T8620","T862
1","T8622",

"T86810","T86811","T86819","T8640","T8641","T8642","T86890","T86891","
T86899","T86850",

"T86851","T86859","T865","T8690","T8691","T8692","T8699","T86890","T86
891","T86899") then transplant_ccc=1;

   end;

   do i=1 to &n_pcs.;

   if pcc(i) in
("0016070","0016071","0016072","0016073","0016074","0016075","0016076"
```

```
,"0016077",

"0016078","001607B","00160J0","00160J1","00160J2","00160J4","00160J5",
"00160J6","00160J7",

"00160J8","00160JB","00160K0","00160K1","00160K2","00160K3","00160K4",
"00160K5","00160K6",

"00160K7","00160K8","00160KB","0016370","0016371","0016372","0016373",
"0016374","0016375",

"0016376","0016377","0016378","001637B","00163J0","00163J1","00163J2",
"00163J3","00163J4",

"00163J5","00163J6","00163J7","00163J8","00163JB","00163K0","00163K1",
"00163K2","00163K3",

"00163K4","00163K5","00163K6","00163K7","00163K8","00163KB","001U074",
"001U076","001U077",

"001U079","001U0J4","001U0J6","001U0J7","001U0J9","001U0K4","001U0K6",
"001U0K7","001U0K9",

"001U374","001U376","001U377","001U379","001U3J4","001U3J6","001U3J7",
"001U3J9","001U3K4",

"001U3K6","001U3K7","001U3K9","009600Z","009630Z","009640Z","00B70ZZ",
"00B73ZZ","00B74ZZ",

"00H00MZ","00H03MZ","00H04MZ","00H60MZ","00H63MZ","00H64MZ","00HE0MZ",
"00HE3MZ","00HE4MZ",

"00HU0MZ","00HU3MZ","00HU4MZ","00HV0MZ","00HV3MZ","00HV4MZ","00T70ZZ",
"00T73ZZ","00T74ZZ",

"00W60JZ","00W63JZ","00W64JZ","00WU0JZ","00WU3JZ","01HY0MZ","01HY3MZ",
"0DH60MZ","0DH63MZ",

"0DH64MZ","0W110J9","0W110JB","0W110JG","0W110JJ","3E1Q38X","3E1Q38Z")
then neuromusc_ccc=1;

   else if pcc(i) in
("02170ZP","02170ZQ","02170ZR","02BK0ZZ","02H40JZ","02H40KZ","02H43JZ"
,"02H44JZ",

"02H44KZ","02H60JZ","02H60KZ","02H63JZ","02H63KZ","02H63MZ","02H64JZ",
"02H64KZ","02H70KZ",

"02H73JZ","02H73KZ","02H73MZ","02H74KZ","02HA0QZ","02HA0RS","02HA0RZ",
"02HA3QZ","02HA3RS",
```

```
"02HA4QZ","02HA4RS","02HK0JZ","02HK0KZ","02HK3JZ","02HK3KZ","02HK3MZ",
"02HK4JZ","02HK4KZ",

"02HL0JZ","02HL0KZ","02HL0MZ","02HL3JZ","02HL3KZ","02HL3MZ","02HL4JZ",
"02HL4KZ","02HL4MZ",

"02HN0JZ","02HN0KZ","02HN0MZ","02HN3JZ","02HN3KZ","02HN3MZ","02HN4JZ",
"02HN4KZ","02HN4MZ",

"02LR0ZT","02LS0ZZ","02LT0ZZ","02NH0ZZ","02RK0JZ","02RL0JZ","02RM0JZ",
"02RP0JZ","02RQ07Z",

"02RQ0JZ","02RR07Z","02RR0JZ","02SP0ZZ","02SW0ZZ","02U70JZ","02UA0JZ",
"02UA3JZ","02UA4JZ",

"02VR0ZT","02WA0JZ","02WA0QZ","02WA0RZ","02WA3QZ","02WA3RZ","02WA4QZ",
"02WA4RZ","02YA0Z0",

"02YA0Z1","02YA0Z2","03HK0MZ","03HK3MZ","03HK4MZ","03HL0MZ","03HL3MZ",
"03HL4MZ","03WY0MZ",

"03WY3MZ","03WY4MZ","0JH600Z","0JH605Z","0JH606Z","0JH607Z","0JH608Z",
"0JH609Z","0JH60AZ",

"0JH60MZ","0JH60PZ","0JH630Z","0JH635Z","0JH636Z","0JH637Z","0JH638Z",
"0JH639Z","0JH63AZ",

"0JH63MZ","0JH63PZ","0JH70MZ","0JH73MZ","0JH800Z","0JH805Z","0JH806Z",
"0JH807Z","0JH808Z",

"0JH809Z","0JH80AZ","0JH80MZ","0JH80PZ","0JH830Z","0JH835Z","0JH836Z",
"0JH837Z","0JH838Z",

"0JH839Z","0JH83AZ","0JH83MZ","0JH83PZ","0JWT0MZ","0JWT0PZ","0JWT3MZ",
"0JWT3PZ","0JWTXMZ",

"4B02XSZ","4B02XTZ","5A02110","5A02116","5A0211D","5A02210","5A02216",
"5A0221D") then cvd_ccc=1;

   else if pcc(i) in:
("0B110F4","0B110Z4","0B113F4","0B113Z4","0B114F4","0B114Z4","0B21XFZ"
,"0BHR0MZ",

"0BHR3MZ","0BHR4MZ","0BHS0MZ","0BHS3MZ","0BHS4MZ","0BTC0ZZ","0BTC4ZZ",
"0BTD0ZZ","0BTD4ZZ",

"0BTF0ZZ","0BTF4ZZ","0BTG0ZZ","0BTG4ZZ","0BTJ0ZZ","0BTJ4ZZ","0BTK0ZZ",
"0BTK4ZZ","0BTL0ZZ",
```

```
"0BTL4ZZ","0BTM0ZZ","0BTM4ZZ","0BW10FZ","0BW13FZ","0BW14FZ","0BYC0Z0",
"0BYC0Z1","0BYC0Z2",

"0BYD0Z0","0BYD0Z1","0BYD0Z2","0BYF0Z0","0BYF0Z1","0BYF0Z2","0BYG0Z0",
"0BYG0Z1","0BYG0Z2",

"0BYH0Z0","0BYH0Z1","0BYH0Z2","0BYJ0Z0","0BYJ0Z1","0BYJ0Z2","0BYK0Z0",
"0BYK0Z1","0BYK0Z2",

"0BYL0Z0","0BYL0Z1","0BYL0Z2","0BYM0Z0","0BYM0Z1","0BYM0Z2","0CTS0ZZ",
"0CTS4ZZ","0CTS7ZZ",

"0CTS8ZZ","0JH604Z","0JH634Z","0JH804Z","0JH834Z","0WQ6XZ2","3E1F78Z")
then respiratory_ccc=1;

   else if pcc(i) in
("031209D","031209F","03120AD","03120AF","03120JD","03120JF","03120KD"
,"03120KF",

"03120ZD","03120ZF","031309D","031309F","03130AD","03130AF","03130JD",
"03130JF","03130KD",

"03130KF","03130ZD","03130ZF","031409D","031409F","03140AD","03140AF",
"03140JD","03140JF",

"03140KD","03140KF","03140ZD","03140ZF","031509D","031509F","03150AD",
"03150AF","03150JF",

"03150KF","03150ZF","031609F","03160AD","03160AF","03160JD","03160KD",
"03160KF","03160ZD",

"03160ZF","031709D","031709F","03170AD","03170AF","03170JD","03170JF",
"03170KD","03170KF",

"03170ZD","03170ZF","031809D","031809F","03180AD","03180AF","03180JD",
"03180JF","03180KD",

"03180KF","03180ZD","03180ZF","031909F","03190AF","03190JF","03190KF",
"03190ZF","031A09F",

"031A0AF","031A0JF","031A0KF","031A0ZF","031B09F","031B0AF","031B0KF",
"031B0ZF","031C09F",

"031C0AF","031C0JF","031C0KF","031C0ZF","03WY0JZ","03WY3JZ","03WY4JZ",
"03WYXJZ","05HY33Z",

"0JH60WZ","0JH60XZ","0JH63WZ","0JH63XZ","0JH80WZ","0JH80XZ","0JH83WZ",
"0JH83XZ","0JHD0XZ",

"0JHD3WZ","0JHD3XZ","0JHF0WZ","0JHF0XZ","0JHF3WZ","0JHF3XZ","0JHL0WZ",
```

"0JHL0XZ","0JHL3WZ",

"0JHL3XZ","0JHM0WZ","0JHM0XZ","0JHM3WZ","0JHM3XZ","0T130ZB","0T134ZB",
"0T140ZB","0T144ZB",

"0T16079","0T1607C","0T160J9","0T160JC","0T160K9","0T160KC","0T160KD",
"0T160Z8","0T160Z9",

"0T160ZA","0T160ZC","0T160ZD","0T163JD","0T16479","0T1647D","0T164J9",
"0T164JC","0T164JD",

"0T164K9","0T164KC","0T164KD","0T164Z8","0T164Z9","0T164ZA","0T164ZC",
"0T164ZD","0T17079",

"0T1707C","0T1707D","0T170J9","0T170JC","0T170JD","0T170K9","0T170KD",
"0T170Z8","0T170Z9",

"0T170ZA","0T170ZC","0T170ZD","0T173JD","0T1747C","0T1747D","0T174J9",
"0T174JC","0T174JD",

"0T174K9","0T174KC","0T174KD","0T174Z8","0T174Z9","0T174ZA","0T174ZC",
"0T174ZD","0T18079",

"0T1807C","0T1807D","0T180J9","0T180JC","0T180JD","0T180K9","0T180KC",
"0T180KD","0T180Z8",

"0T180Z9","0T180ZA","0T180ZC","0T180ZD","0T18479","0T1847C","0T1847D",
"0T184J9","0T184JC",

"0T184JD","0T184K9","0T184KC","0T184KD","0T184Z8","0T184Z9","0T184ZA",
"0T184ZC","0T184ZD",

"0T1B0ZD","0T1B4ZD","0T25X0Z","0T29X0Z","0T29XYZ","0T2BX0Z","0T9000Z",
"0T9030Z","0T9040Z",

"0T9070Z","0T9080Z","0T9100Z","0T9130Z","0T9140Z","0T9170Z","0T9180Z",
"0T9370Z","0T9380Z",

"0T9470Z","0T9480Z","0TB60ZZ","0TB63ZZ","0TB64ZZ","0TB67ZZ","0TB68ZZ",
"0TB70ZZ","0TB74ZZ",

"0TB77ZZ","0TB78ZZ","0TQ67ZZ","0TQ77ZZ","0TT00ZZ","0TT04ZZ","0TT10ZZ",
"0TT14ZZ","0TT20ZZ",

"0TT24ZZ","0TT60ZZ","0TT64ZZ","0TT67ZZ","0TT68ZZ","0TT70ZZ","0TT74ZZ",
"0TT77ZZ","0TT78ZZ",

"0TTB0ZZ","0TTB4ZZ","0TTB7ZZ","0TTB8ZZ","0TTD0ZZ","0TTD4ZZ","0TTD7ZZ",
"0TTD8ZZ","0TY00Z0",

```
"0TY00Z1","0TY00Z2","0TY10Z0","0TY10Z1","0TY10Z2","3E1K38Z","3E1M39Z",
"5A1D60Z") then renal_ccc=1;

   else if pcc(i) in
("0CT70ZZ","0CT7XZZ","0D11074","0D110J4","0D110K4","0D110Z4","0D113J4"
,"0D11474",

"0D114J4","0D114K4","0D114Z4","0D13079","0D1307A","0D1307B","0D15074",
"0D150J4","0D150K4",

"0D150Z4","0D153J4","0D15474","0D154J4","0D154K4","0D154Z4","0D16074",
"0D1607A","0D160J4",

"0D160J9","0D160JA","0D160K4","0D160K9","0D160KA","0D160Z4","0D160ZA",
"0D163J4","0D16474",

"0D164J4","0D164J9","0D164JA","0D164K4","0D164K9","0D164KA","0D164Z4",
"0D16874","0D168J4",

"0D168J9","0D168JA","0D168K4","0D168K9","0D168KA","0D168Z4","0D1B0Z4",
"0D1B4Z4","0D1B8Z4",

"0D1H0Z4","0D1H4Z4","0D1H8Z4","0D1K0Z4","0D1K4Z4","0D1K8Z4","0D1L0Z4",
"0D1L4Z4","0D1L8Z4",

"0D1N0Z4","0D1N4Z4","0D1N8Z4","0D20X0Z","0D20XUZ","0D20XYZ","0D787ZZ",
"0D7E7ZZ","0DBB7ZZ",

"0DH50DZ","0DH50UZ","0DH53DZ","0DH53UZ","0DH54DZ","0DH54UZ","0DH57DZ",
"0DH57UZ","0DH58DZ",

"0DH58UZ","0DH63UZ","0DH64UZ","0DHA3UZ","0DHA4UZ","0DHA8UZ","0DN87ZZ",
"0DNE7ZZ","0DT50ZZ",

"0DT54ZZ","0DT57ZZ","0DT58ZZ","0DT60ZZ","0DT64ZZ","0DT67ZZ","0DT68ZZ",
"0DT80ZZ","0DT84ZZ",

"0DT87ZZ","0DT88ZZ","0DT90ZZ","0DT94ZZ","0DT97ZZ","0DT98ZZ","0DTE0ZZ",
"0DTE4ZZ","0DTE7ZZ",

"0DTE8ZZ","0DW04UZ","0DW08UZ","0DY80Z0","0DY80Z1","0DY80Z2","0DYE0Z0",
"0DYE0Z1","0DYE0Z2",

"0FT00ZZ","0FT04ZZ","0FTG0ZZ","0FTG4ZZ","0FY00Z0","0FY00Z1","0FY00Z2",
"0FYG0Z0","0FYG0Z1",

"0FYG0Z2","0WQFXZ2","3E030U0","3E030U1","3E033U0","3E033U1","3E0J3U0",
"3E0J3U1","3E0J7U0",
          "3E0J7U1","3E0J8U0","3E0J8U1","3E1G78Z","3E1H78Z") then
GI_ccc=1;
```

```
   else if pcc(i) in
("07TP0ZZ","07TP4ZZ","07YP0Z0","07YP0Z1","07YP0Z2","30230AZ","30230G0"
,"30230G1",

"30230X0","30230X1","30230Y0","30230Y1","30233AZ","30233G0","30233G1",
"30233X0","30233X1",

"30233Y0","30233Y1","30240AZ","30240G0","30240G1","30240X0","30240X1",
"30240Y0","30240Y1",

"30243AZ","30243G0","30243G1","30243X0","30243X1","30243Y0","30243Y1",
"30250G0","30250G1",

"30250X0","30250X1","30250Y0","30250Y1","30253G0","30253G1","30253X0",
"30253X1","30253Y0",

"30253Y1","30260G0","30260G1","30260X0","30260X1","30260Y0","30260Y1",
"30263G0","30263G1",
          "30263X0","30263X1","30263Y0","30263Y1") then
hemato_immu_ccc=1;

   else if pcc(i) in
("0GT00ZZ","0GT04ZZ","0GT40ZZ","0GT44ZZ","0GTK0ZZ","0GTK4ZZ","0GTR0ZZ"
,"0GTR4ZZ",

"0JH60VZ","0JH63VZ","0JH70VZ","0JH73VZ","0JH80VZ","0JH83VZ","0JHD0VZ",
"0JHD3VZ","0JHF0VZ",

"0JHF3VZ","0JHG0VZ","0JHG3VZ","0JHH0VZ","0JHH3VZ","0JHL0VZ","0JHL3VZ",
"0JHM0VZ","0JHM3VZ",

"0JHN0VZ","0JHN3VZ","0JHP0VZ","0JHP3VZ","0JHT0VZ","0JHT3VZ","0UT20ZZ",
"0UT24ZZ","0UT27ZZ",

"0UT28ZZ","0UT2FZZ","0UT40ZZ","0UT44ZZ","0UT47ZZ","0UT48ZZ","0UT70ZZ",
"0UT74ZZ","0UT90ZZ",

"0UT94ZZ","0UT97ZZ","0UT98ZZ","0UT9FZZ","0UTC0ZZ","0UTC7ZZ","0UTC8ZZ",
"0VTC0ZZ","0VTC4ZZ",

"0W4M070","0W4M0J0","0W4M0K0","0W4M0Z0","0W4N071","0W4N0J1","0W4N0K1",
"0W4N0Z1") then metabolic_ccc=1;

   else if pcc(i) in
("3E00X05","3E01305","3E02305","3E03005","3E03305","3E04005","3E04305"
,"3E05005",

"3E05305","3E06005","3E06305","3E0A305","3E0F305","3E0F705","3E0F805",
"3E0G305","3E0G705",
```

```
"3E0G805","3E0H305","3E0H705","3E0H805","3E0J305","3E0J705","3E0J805",
"3E0K305","3E0K705",

"3E0K805","3E0L305","3E0L705","3E0M305","3E0M705","3E0N305","3E0N705",
"3E0N805","3E0P305",

"3E0P705","3E0P805","3E0Q305","3E0Q705","3E0R305","3E0S305","3E0V305",
"3E0W305","3E0Y305",
          "3E0Y705") then malignancy_ccc=1;

   if pcc(i) in
("00160J0","00160J1","00160J2","00160J4","00160J5","00160J6","00160J7"
,"00160J8",

"00160JB","00160K0","00160K1","00160K2","00160K3","00160K4","00160K5",
"00160K6","00160K7",

"00160K8","00160KB","00163J0","00163J1","00163J2","00163J3","00163J4",
"00163J5","00163J6",

"00163J7","00163J8","00163JB","00163K0","00163K1","00163K2","00163K3",
"00163K4","00163K5",

"00163K6","00163K7","00163K8","00163KB","001U0J4","001U0J6","001U0J7",
"001U0J9","001U0K4",

"001U0K6","001U0K7","001U0K9","001U3J4","001U3J6","001U3J7","001U3J9",
"001U3K4","001U3K6",

"001U3K7","001U3K9","009600Z","009630Z","009640Z","00H00MZ","00H03MZ",
"00H04MZ","00H60MZ",

"00H63MZ","00H64MZ","00HE0MZ","00HE3MZ","00HE4MZ","00HU0MZ","00HU3MZ",
"00HU4MZ","00HV0MZ",

"00HV3MZ","00HV4MZ","00W60JZ","00W63JZ","00W64JZ","00WU0JZ","00WU3JZ",
"01HY0MZ","01HY3MZ",

"0DH60MZ","0DH63MZ","0DH64MZ","0W110J9","0W110JB","0W110JG","0W110JJ",
"3E1Q38X","3E1Q38Z",

"02H40JZ","02H40KZ","02H43JZ","02H44JZ","02H44KZ","02H60JZ","02H60KZ",
"02H63JZ","02H63KZ",

"02H63MZ","02H64JZ","02H64KZ","02H70KZ","02H73JZ","02H73KZ","02H73MZ",
"02H74KZ","02HA0QZ",

"02HA0RS","02HA0RZ","02HA3QZ","02HA3RS","02HA4QZ","02HA4RS","02HK0JZ",
"02HK0KZ","02HK3JZ",
```

"02HK3KZ","02HK3MZ","02HK4JZ","02HK4KZ","02HL0JZ","02HL0KZ","02HL0MZ",
"02HL3JZ","02HL3KZ",

"02HL3MZ","02HL4JZ","02HL4KZ","02HL4MZ","02HN0JZ","02HN0KZ","02HN0MZ",
"02HN3JZ","02HN3KZ",

"02HN3MZ","02HN4JZ","02HN4KZ","02HN4MZ","02WA0QZ","02WA0RZ","02WA3QZ",
"02WA3RZ","02WA4QZ",

"02WA4RZ","03HK0MZ","03HK3MZ","03HK4MZ","03HL0MZ","03HL3MZ","03HL4MZ",
"03WY0MZ","03WY3MZ",

"03WY4MZ","0JH600Z","0JH605Z","0JH606Z","0JH607Z","0JH608Z","0JH609Z",
"0JH60AZ","0JH60MZ",

"0JH60PZ","0JH630Z","0JH635Z","0JH636Z","0JH637Z","0JH638Z","0JH639Z",
"0JH63AZ","0JH63MZ",

"0JH63PZ","0JH70MZ","0JH73MZ","0JH800Z","0JH805Z","0JH806Z","0JH807Z",
"0JH808Z","0JH809Z",

"0JH80AZ","0JH80MZ","0JH80PZ","0JH830Z","0JH835Z","0JH836Z","0JH837Z",
"0JH838Z","0JH839Z",

"0JH83AZ","0JH83MZ","0JH83PZ","0JWT0MZ","0JWT0PZ","0JWT3MZ","0JWT3PZ",
"0JWTXMZ","4B02XSZ",

"4B02XTZ","5A02110","5A02116","5A0211D","5A02210","5A02216","5A0221D",
"0B110F4","0B113F4",

"0B114F4","0B21XFZ","0BHR0MZ","0BHR3MZ","0BHR4MZ","0BHS0MZ","0BHS3MZ",
"0BHS4MZ","0BW10FZ",

"0BW13FZ","0BW14FZ","0JH604Z","0JH634Z","0JH804Z","0JH834Z","0WQ6XZ2",
"3E1F78Z","031209D",

"031209F","03120AD","03120AF","03120JD","03120JF","03120KD","03120KF",
"03120ZD","03120ZF",

"031309D","031309F","03130AD","03130AF","03130JD","03130JF","03130KD",
"03130KF","03130ZD",

"03130ZF","031409D","031409F","03140AD","03140AF","03140JD","03140JF",
"03140KD","03140KF",

"03140ZD","03140ZF","031509D","031509F","03150AD","03150AF","03150JF",
"03150KF","03150ZF",

"031609F","03160AD","03160AF","03160JD","03160KD","03160KF","03160ZD",

"03160ZF","031709D",

"031709F","03170AD","03170AF","03170JD","03170JF","03170KD","03170KF",
"03170ZD","03170ZF",

"031809D","031809F","03180AD","03180AF","03180JD","03180JF","03180KD",
"03180KF","03180ZD",

"03180ZF","031909F","03190AF","03190JF","03190KF","03190ZF","031A09F",
"031A0AF","031A0JF",

"031A0KF","031A0ZF","031B09F","031B0AF","031B0KF","031B0ZF","031C09F",
"031C0AF","031C0JF",

"031C0KF","031C0ZF","03WY0JZ","03WY3JZ","03WY4JZ","03WYXJZ","05HY33Z",
"0JH60WZ","0JH60XZ",

"0JH63WZ","0JH63XZ","0JH80WZ","0JH80XZ","0JH83WZ","0JH83XZ","0JHD0XZ",
"0JHD3WZ","0JHD3XZ",

"0JHF0WZ","0JHF0XZ","0JHF3WZ","0JHF3XZ","0JHL0WZ","0JHL0XZ","0JHL3WZ",
"0JHL3XZ","0JHM0WZ",

"0JHM0XZ","0JHM3WZ","0JHM3XZ","0T130ZB","0T134ZB","0T140ZB","0T144ZB",
"0T16079","0T1607C",

"0T160J9","0T160JC","0T160K9","0T160KC","0T160KD","0T160Z9","0T160ZC",
"0T160ZD","0T163JD",

"0T16479","0T1647D","0T164J9","0T164JC","0T164JD","0T164K9","0T164KC",
"0T164KD","0T164Z9",

"0T164ZC","0T164ZD","0T17079","0T1707C","0T1707D","0T170J9","0T170JC",
"0T170JD","0T170K9",

"0T170KD","0T170Z9","0T170ZC","0T170ZD","0T173JD","0T1747C","0T1747D",
"0T174J9","0T174JC",

"0T174JD","0T174K9","0T174KC","0T174KD","0T174Z9","0T174ZC","0T174ZD",
"0T18079","0T1807C",

"0T1807D","0T180J9","0T180JC","0T180JD","0T180K9","0T180KC","0T180KD",
"0T180Z9","0T180ZC",

"0T180ZD","0T18479","0T1847C","0T1847D","0T184J9","0T184JC","0T184JD",
"0T184K9","0T184KC",

"0T184KD","0T184Z9","0T184ZC","0T184ZD","0T1B0ZD","0T1B4ZD","0T25X0Z",
"0T29X0Z","0T29XYZ",

"0T2BX0Z","0T9000Z","0T9030Z","0T9040Z","0T9070Z","0T9080Z","0T9100Z",
"0T9130Z","0T9140Z",

"0T9170Z","0T9180Z","0T9370Z","0T9380Z","0T9470Z","0T9480Z","0TQ67ZZ",
"0TQ77ZZ","3E1K38Z",

"3E1M39Z","5A1D60Z","0D11074","0D110J4","0D110K4","0D110Z4","0D113J4",
"0D11474","0D114J4",

"0D114K4","0D114Z4","0D15074","0D150J4","0D150K4","0D150Z4","0D153J4",
"0D15474","0D154J4",

"0D154K4","0D154Z4","0D16074","0D160J4","0D160J9","0D160JA","0D160K4",
"0D160K9","0D160KA",

"0D160Z4","0D163J4","0D16474","0D164J4","0D164J9","0D164JA","0D164K4",
"0D164K9","0D164KA",

"0D164Z4","0D16874","0D168J4","0D168J9","0D168JA","0D168K4","0D168K9",
"0D168KA","0D168Z4",

"0D1B0Z4","0D1B4Z4","0D1B8Z4","0D1H0Z4","0D1H4Z4","0D1H8Z4","0D1K0Z4",
"0D1K4Z4","0D1K8Z4",

"0D1L0Z4","0D1L4Z4","0D1L8Z4","0D1N0Z4","0D1N4Z4","0D1N8Z4","0D20X0Z",
"0D20XUZ","0D20XYZ",

"0D787ZZ","0D7E7ZZ","0DBB7ZZ","0DH50DZ","0DH50UZ","0DH53DZ","0DH53UZ",
"0DH54DZ","0DH54UZ",

"0DH57DZ","0DH57UZ","0DH58DZ","0DH58UZ","0DH63UZ","0DH64UZ","0DHA3UZ",
"0DHA4UZ","0DHA8UZ",

"0DN87ZZ","0DNE7ZZ","0DW04UZ","0DW08UZ","0WQFXZ2","3E1G78Z","3E1H78Z",
"0JH60VZ","0JH63VZ",

"0JH70VZ","0JH73VZ","0JH80VZ","0JH83VZ","0JHD0VZ","0JHD3VZ","0JHF0VZ",
"0JHF3VZ","0JHG0VZ",

"0JHG3VZ","0JHH0VZ","0JHH3VZ","0JHL0VZ","0JHL3VZ","0JHM0VZ","0JHM3VZ",
"0JHN0VZ","0JHN3VZ",

"0JHP0VZ","0JHP3VZ","0JHT0VZ","0JHT3VZ","0RG00J0","0RG00J1","0RG00JJ",
"0RG00K0","0RG00K1",

"0RG00KJ","0RG00Z0","0RG00Z1","0RG00ZJ","0RG03J0","0RG03J1","0RG03JJ",
"0RG03K0","0RG03K1",

"0RG03KJ","0RG03Z0","0RG03Z1","0RG03ZJ","0RG04J0","0RG04J1","0RG04JJ",
"0RG04K0","0RG04K1",

```
"0RG04KJ","0RG04Z0","0RG04Z1","0RG04ZJ","0RG10J0","0RG10J1","0RG10JJ",
"0RG10K0","0RG10K1",

"0RG10KJ","0RG10Z0","0RG10Z1","0RG10ZJ","0RG13J0","0RG13J1","0RG13JJ",
"0RG13K0","0RG13K1",

"0RG13KJ","0RG13Z0","0RG13Z1","0RG13ZJ","0RG14J0","0RG14J1","0RG14JJ",
"0RG14K0","0RG14K1",

"0RG14KJ","0RG14Z0","0RG14Z1","0RG14ZJ","0RG40J0","0RG40J1","0RG40JJ",
"0RG40K0","0RG40K1",

"0RG40KJ","0RG40Z0","0RG40Z1","0RG40ZJ","0RG43J0","0RG43J1","0RG43JJ",
"0RG43K0","0RG43K1",

"0RG43KJ","0RG43Z0","0RG43Z1","0RG43ZJ","0RG44J0","0RG44J1","0RG44JJ",
"0RG44K0","0RG44K1",

"0RG44KJ","0RG44Z0","0RG44Z1","0RG44ZJ","0RG60J0","0RG60J1","0RG60JJ",
"0RG60K0","0RG60K1",

"0RG60KJ","0RG60Z0","0RG60Z1","0RG60ZJ","0RG63J0","0RG63J1","0RG63JJ",
"0RG63K0","0RG63K1",

"0RG63KJ","0RG63Z0","0RG63Z1","0RG63ZJ","0RG64J0","0RG64J1","0RG64JJ",
"0RG64K0","0RG64K1",

"0RG64KJ","0RG64Z0","0RG64Z1","0RG64ZJ","0RGA0J0","0RGA0J1","0RGA0JJ",
"0RGA0K0","0RGA0K1",

"0RGA0KJ","0RGA0Z0","0RGA0Z1","0RGA0ZJ","0RGA3J0","0RGA3J1","0RGA3JJ",
"0RGA3K0","0RGA3K1",

"0RGA3KJ","0RGA3Z0","0RGA3Z1","0RGA3ZJ","0RGA4J0","0RGA4J1","0RGA4JJ",
"0RGA4K0","0RGA4K1",

"0RGA4KJ","0RGA4Z0","0RGA4Z1","0RGA4ZJ","0SG00J0","0SG00J1","0SG00JJ",
"0SG00K0","0SG00K1",

"0SG00KJ","0SG00Z0","0SG00Z1","0SG00ZJ","0SG03J0","0SG03J1","0SG03JJ",
"0SG03K0","0SG03K1",

"0SG03KJ","0SG03Z0","0SG03Z1","0SG03ZJ","0SG04J0","0SG04J1","0SG04JJ",
"0SG04K0","0SG04K1",
          "0SG04KJ","0SG04Z0","0SG04Z1","0SG04ZJ") then
tech_dep_ccc=1;

   if pcc(i) in
("02YA0Z0","02YA0Z1","02YA0Z2","0BYC0Z0","0BYC0Z1","0BYC0Z2","0BYD0Z0"
```

```
,"0BYD0Z1",

"0BYD0Z2","0BYF0Z0","0BYF0Z1","0BYF0Z2","0BYG0Z0","0BYG0Z1","0BYG0Z2",
"0BYH0Z0","0BYH0Z1",

"0BYH0Z2","0BYJ0Z0","0BYJ0Z1","0BYJ0Z2","0BYK0Z0","0BYK0Z1","0BYK0Z2",
"0BYL0Z0","0BYL0Z1",

"0BYL0Z2","0BYM0Z0","0BYM0Z1","0BYM0Z2","0TY00Z0","0TY00Z1","0TY00Z2",
"0TY10Z0","0TY10Z1",

"0TY10Z2","0DY80Z0","0DY80Z1","0DY80Z2","0DYE0Z0","0DYE0Z1","0DYE0Z2",
"0FY00Z0","0FY00Z1",

"0FY00Z2","0FYG0Z0","0FYG0Z1","0FYG0Z2","3E030U0","3E030U1","3E033U0",
"3E033U1","3E0J3U0",

"3E0J3U1","3E0J7U0","3E0J7U1","3E0J8U0","3E0J8U1","07YP0Z0","07YP0Z1",
"07YP0Z2","30230AZ",

"30230G0","30230G1","30230X0","30230X1","30230Y0","30230Y1","30233AZ",
"30233G0","30233G1",

"30233X0","30233X1","30233Y0","30233Y1","30240AZ","30240G0","30240G1",
"30240X0","30240X1",

"30240Y0","30240Y1","30243AZ","30243G0","30243G1","30243X0","30243X1",
"30243Y0","30243Y1",

"30250G0","30250G1","30250X0","30250X1","30250Y0","30250Y1","30253G0",
"30253G1","30253X0",

"30253X1","30253Y0","30253Y1","30260G0","30260G1","30260X0","30260X1",
"30260Y0","30260Y1",
          "30263G0","30263G1","30263X0","30263X1","30263Y0","30263Y1")
then transplant_ccc=1;

   end;

   end;

   drop i;


num_ccc=sum(neuromusc_ccc,cvd_ccc,respiratory_ccc,renal_ccc,GI_ccc,hem
ato_immu_ccc,

metabolic_ccc,congeni_genetic_ccc,malignancy_ccc,neonatal_ccc);
   ccc_flag=num_ccc>0;
```

```
    if transplant_ccc=1 then ccc_flag=1;
    if tech_dep_ccc=1 then ccc_flag=1;

run;
%mend ccc_version2_sas;
```
