# Supplementary material for: Pediatric complex chronic conditions classification system version 2: updated for ICD-10 and complex medical technology dependence and transplantation
Source: BMC Pediatr. 2014 Aug 8;14:199. doi: 10.1186/1471-2431-14-199 (PMC4134331; doi:10.1186/1471-2431-14-199)
Supplement: Additional file 4 — CCC V2 Stata Program. PDF Stata macro program code to apply the CCC V2 system to user data. [file 1471-2431-14-199-S4.pdf]

```
/****************************************************************************
| Program Name: ccc                                                         |
|                                                                           |
|***************************************************************************|
| Function:                                                                 |
|                                                                           |
| This program utilizes ICD-9-CM or ICD-10 diagnostic and procedural codes  |
| to create variables representing the presence of a CCC, the type of CCC,  |
| technology/device usage, and transplantation status based on the articles:|
|                                                                           |
| Complex Chronic Conditions Version 2: Feudtner C, et al.                  |
|                                                                           |
|****************************************************************************
| Author: James Feinstein MD MPH                                            |
| Creation Date: 2013-08-19                                                 |
| Revision Date: 2014-04-08                                                 |
|                                                                           |
|****************************************************************************
| Program call statement:                                                   |
| ccc dt_out dx n_dxs pc n_pcs icdv                                         |
|                                                                           |
| NOTE: The variables containing the ICD codes MUST follow the naming       |
| convention of a prefix followed by sequential numbers, i.e. dx1, dx2, dx3 |
|                                                                           |
| Parameter definitions:                                                    |
|                                                                           |
|  dt_out:     File name for output data; will be annoted with "_ccc"       |
|                                                                           |
|  dx:         ICD-9-CM or ICD-10 variable prefix name following naming     |
|              convention prefix# (i.e. dx1, dx2, ...)                      |
|                                                                           |
|  n_dxs:      Number of ICD-9-CM or ICD-10 variables in data set           |
|                                                                           |
|  pc:         ICD-9-CM procedure or ICD-10 procedure variable prefix name  |
|              following naming convention prefix# (i.e. pc1, pc2, ...)     |
|                                                                           |
|  n_pcs:      Number of ICD-9-CM or ICD-10 procedure variables in data set |
```

```
|                                                                              |
|  icdv:       ICD revision 9 or 10                                            |
|                                                                              |
|******************************************************************************|
|  Examples:                                                                   |
|                                                                              |
| 1. Apply CCC v2 to array of ICD-9-CM diagnostic and procedural codes         |
|    named dx1-dx5 and px1-px3:                                                |
|                                                                              |
|     ccc output_file_name dx 5 px 3 9                                         |
|                                                                              |
| 2. Apply CCC v2 to array of ICD-10 diagnostic and procedural codes           |
|    named diag1-diag10 and proc1-proc7:                                       |
|                                                                              |
|    ccc output_file_name diag 10 proc 7 10                                    |
|                                                                              |
******************************************************************************/


* Define program
program define ccc
       args dt_out dx n_dxs pc n_pcs icdv

set more off

* Create categories of CCCs to match SAS coded variables
local ccc_class neuromusc_ccc cvd_ccc respiratory_ccc renal_ccc gi_ccc ///
       hemato_immu_ccc metabolic_ccc congeni_genetic_ccc malignancy_ccc ///
       neonatal_ccc tech_dep_ccc transplant_ccc

foreach  x of local ccc_class {
       gen `x'=0
       }

* Prepare ICD9/10 variables for search sequence by trimming leading and
* trailing blank spaces
```

```
forvalues i=1/`n_dxs' {
	replace `dx'`i' = trim(`dx'`i')
	icd9 clean `dx'`i'
	}

forvalues i=1/`n_pcs' {
	replace `pc'`i' = trim(`pc'`i')
	icd9p clean `dx'`i'
	}

* Create temporary count variables
gen id = [_n]
gen dx_count = 0
gen pc_count = 0

forvalues i=1/`n_dxs' {
	replace dx_count = dx_count + 1 if `dx'`i'!=""
	}

forvalues i=1/`n_pcs' {
	replace pc_count = pc_count + 1 if `pc'`i'!=""
	}

*Run search sequence for CCC Version 2

if `icdv'==9 {

* ANALYZE ICD-9 DIAGNOSTIC CODES

gsort -dx_count

forvalues i=1/`n_dxs' {

if `dx'`i' !="" {

* NEUROMUSCULAR CCC ICD-9
```

```
local nm_icd_3 330 334 335 343 359 740 741 742

local nm_icd_4 3180 3181 3182 3311 3314 3318 3319 3320 3321 3330 ///

       3332 3334 3335 3337 3339 3590 3591 3592 3593 3361 3368 3379 ///

       3418 3440 3449 3453 3481 3484 7595 3491 9962 V452

local nm_icd_5 33111 33119 33189 34290 34481 34501 34511 34541 ///

       34561 34571 34581 34591 43401 43491 78003 99663 V5301 V5302


       foreach nm_x of local nm_icd_3 {

              replace neuromusc_ccc = 1 if substr(`dx'`i',1,3)=="`nm_x'"

              }

       foreach nm_y of local nm_icd_4 {

              replace neuromusc_ccc = 1 if substr(`dx'`i',1,4)=="`nm_y'"

              }

       foreach nm_z of local nm_icd_5 {

              replace neuromusc_ccc = 1 if substr(`dx'`i',1,5)=="`nm_z'"

              }


* CARDIOVASCULAR CCC ICD-9


local cv_icd_3 416 426 746

local cv_icd_4 4161 4168 4169 4240 4242 4243 4250 4251 4252 4253 ///

       4254 4258 4270 4271 4272 4273 4274 4276 4277 4278 4279 4280 ///

       42883 4291 4293 7450 7451 7452 7453 7456 7471 7472 7473 ///

       7474 9960 9961 V421 V422 V432 V433 V450 V533

local cv_icd_5 43311 74781 74789 99661 99662 V4581


       foreach cv_x of local cv_icd_3 {

              replace cvd_ccc = 1 if substr(`dx'`i',1,3)=="`cv_x'"

              }

       foreach cv_y of local cv_icd_4 {

              replace cvd_ccc = 1 if substr(`dx'`i',1,4)=="`cv_y'"

              }

       foreach cv_z of local cv_icd_5 {

              replace cvd_ccc = 1 if substr(`dx'`i',1,5)=="`cv_z'"

              }
```

```
* RESPIRATORY CCC ICD-9

local rp_icd_3 748
local rp_icd_4 2770 4160 4162 5163 5190 7704 V426 V440 V460 V461 V550
local rp_icd_5 V4576 32725 51631 51884

	foreach rp_x of local rp_icd_3 {
		replace respiratory_ccc = 1 if substr(`dx'`i',1,3)=="`rp_x'"
		}
	foreach rp_y of local rp_icd_4 {
		replace respiratory_ccc = 1 if substr(`dx'`i',1,4)=="`rp_y'"
		}
	foreach rp_z of local rp_icd_5 {
		replace respiratory_ccc = 1 if substr(`dx'`i',1,5)=="`rp_z'"
		}

* RENAL CCC ICD-9

local rn_icd_3 585 753 V56
local rn_icd_4 5964 V420 V445 V446 V451 V536 V555 V556
local rn_icd_5 34461 59653 59654 99668 V4573 V4574

	foreach rn_x of local rn_icd_3 {
		replace renal_ccc = 1 if substr(`dx'`i',1,3)=="`rn_x'"
		}
	foreach rn_y of local rn_icd_4 {
		replace renal_ccc = 1 if substr(`dx'`i',1,4)=="`rn_y'"
		}
	foreach rn_z of local rn_icd_5 {
		replace renal_ccc = 1 if substr(`dx'`i',1,5)=="`rn_z'"
		}

* GI CCC ICD-9

local gi_icd_3 555 556 751
local gi_icd_4 4530 5364 5571 5602 5647 5714 5715 5716 5717 5718 5719 ///
```

```
        7503 V427 V441 V442 V443 V444 V551 V552 V553 V554
local gi_icd_5 V4283 V4284 V5350 V5351 V5359


        foreach gi_x of local gi_icd_3 {
                replace gi_ccc = 1 if substr(`dx'`i',1,3)=="`gi_x'"
                }
        foreach gi_y of local gi_icd_4 {
                replace gi_ccc = 1 if substr(`dx'`i',1,4)=="`gi_y'"
                }
        foreach gi_z of local gi_icd_5 {
                replace gi_ccc = 1 if substr(`dx'`i',1,5)=="`gi_z'"
                }

* HEME/IMMUNO CCC ICD-9

local hi_icd_3 042 043 044 135 279 284 V08
local hi_icd_4 2820 2821 2822 2823 2824 2825 2826 2881 2882 2860 2863 ///
        2884 4460 4461 4464 4465 4466 4467 7100 7101 7103
local hi_icd_5 28732 28733 28739 28801 28802 44621

        foreach hi_x of local hi_icd_3 {
                replace hemato_immu_ccc = 1 if substr(`dx'`i',1,3)=="`hi_x'"
                }
        foreach hi_y of local hi_icd_4 {
                replace hemato_immu_ccc = 1 if substr(`dx'`i',1,4)=="`hi_y'"
                }
        foreach hi_z of local hi_icd_5 {
                replace hemato_immu_ccc = 1 if substr(`dx'`i',1,5)=="`hi_z'"
                }

* METABOLIC CCC ICD-9

local mb_icd_3 243 270 271 272
local mb_icd_4 2532 2535 2536 2359 2550 2552 2750 2751 2752 2753 2772 ///
        2773 2774 2775 2776 2778 2779 7751
```

```
local mb_icd_5 25513 V4585 V5391 V6546

        foreach mb_x of local mb_icd_3 {
                replace metabolic_ccc = 1 if substr(`dx'`i',1,3)=="`mb_x'"
                }
        foreach mb_y of local mb_icd_4 {
                replace metabolic_ccc = 1 if substr(`dx'`i',1,4)=="`mb_y'"
                }
        foreach mb_z of local mb_icd_5 {
                replace metabolic_ccc = 1 if substr(`dx'`i',1,5)=="`mb_z'"
                }

* CONGENITAL/GENETIC CCC ICD-9

local cg_icd_3 758
local cg_icd_4 2594 5533 7373 7560 7561 7562 7563 7564 7565 7566 7567 ///
        7597 7598 7599

        foreach cg_x of local cg_icd_3 {
                replace congeni_genetic_ccc = 1 if substr(`dx'`i',1,3)=="`cg_x'"
                }
        foreach cg_y of local cg_icd_4 {
                replace congeni_genetic_ccc = 1 if substr(`dx'`i',1,4)=="`cg_y'"
                }

* MALIGNANCY CCC ICD-9

local mg_icd_2 14 15 16 17 18 19 20 23
local mg_icd_5 V4281 V4282

        foreach mg_x of local mg_icd_2 {
                replace malignancy_ccc = 1 if substr(`dx'`i',1,2)=="`mg_x'"
                }
        foreach mg_y of local mg_icd_5 {
                replace malignancy_ccc = 1 if substr(`dx'`i',1,5)=="`mg_y'"
                }
```

```
* NEONATAL CCC ICD-9

local nn_icd_4 7670 7674 7685 7687 7689 7702 7707 7710 7711 7733 ///
       7734 7747 7765 7780 7797
local nn_icd_5 76401 76402 76411 76412 76421 76422 76491 76492 76501 ///
       76502 76511 76512 76521 76522 76523 77213 77214 77753

       foreach nn_x of local nn_icd_4 {
              replace neonatal_ccc = 1 if substr(`dx'`i',1,4)=="`nn_x'"
              }
       foreach nn_y of local nn_icd_5 {
              replace neonatal_ccc = 1 if substr(`dx'`i',1,5)=="`nn_y'"
              }

* TECHNOLOGY DEPENDENT CCC ICD-9

local td_icd_4 5190 5364 9960 9961 9962 9964 9969 V433 V440 ///
       V441 V442 V443 V444 V445 V446 V450 V451 V452 V460 V461 V462 ///
       V536 V550 V551 V552 V553 V554 V555 V556
local td_icd_5 99661 99662 99663 99666 99667 99668 V5301 V5302 ///
       V5331 V5332 V5339 V5350 V5351 V5359

       foreach td_x of local td_icd_4 {
              replace tech_dep_ccc = 1 if substr(`dx'`i',1,4)=="`td_x'"
              }
       foreach td_y of local td_icd_5 {
              replace tech_dep_ccc = 1 if substr(`dx'`i',1,5)=="`td_y'"
              }

* TRANSPLANT CCC ICD-9

local tx_icd_4 V421 V422 V426 V427 V432
local tx_icd_5 99680 99681 99682 99683 99684 99685 99686 99687 99688 ///
       99689 V4281 V4282 V4283 V4284 V4585 V5391

       foreach tx_x of local tx_icd_4 {
```

```
                replace transplant_ccc = 1 if substr(`dx'`i',1,4)=="`tx_x'"
                }
        foreach tx_y of local tx_icd_5 {
                replace transplant_ccc = 1 if substr(`dx'`i',1,5)=="`tx_y'"
                }
}
}

* ANALYZE ICD-9 PROCEDURE CODES

gsort -pc_count

forvalues i=1/`n_pcs' {

if `pc'`i' !="" {

* NEUROMUSCULAR CCC ICD-9

local nm_icd_4 0152 0153 0221 0222 0231 0232 0233 0234 0235 0239 0241 ///
        0242 0293 0371 0372 0379 0393 0397 0492

        foreach nm_y of local nm_icd_4 {
                replace neuromusc_ccc = 1 if substr(`pc'`i',1,4)=="`nm_y'"
                }

* CARDIOVASCULAR CCC ICD-9

local cv_icd_4 0050 0051 0053 0054 0055 0057 1751 1752 3581 3582 3583 ///
        3584 3741 3751 3752 3753 3754 3755 3760 3761 3763 3765 3766 ///
        3767 3768 3771 3772 3774 3776 3779 3780 3781 3782 3783 3785 ///
        3786 3787 3789 3794 3795 3796 3797 3798 3981 3982 3983 3984 ///
        3985 8945 8946 8947 8948 8949

        foreach cv_y of local cv_icd_4 {
                replace cvd_ccc = 1 if substr(`pc'`i',1,4)=="`cv_y'"
                }
```

```
* RESPIRATORY CCC ICD-9

local rp_icd_3 303 304 336
local rp_icd_4 3121 3129 3141 3174 3241 3249 3250 3259 3321 3350 ///
	3351 3352 3485 9655 9723

	foreach rp_x of local rp_icd_3 {
		replace respiratory_ccc = 1 if substr(`pc'`i',1,3)=="`rp_x'"
		}
	foreach rp_y of local rp_icd_4 {
		replace respiratory_ccc = 1 if substr(`pc'`i',1,4)=="`rp_y'"
		}

* RENAL CCC ICD-9

local rn_icd_4 3895 3927 3942 3993 3994 3995 5498 5502 5503 5504 5512 ///
	5551 5552 5553 5554 5561 5569 5593 5594 5597 5641 5642 5651 5652 ///
	5661 5662 5671 5672 5673 5674 5675 5679 5721 5722 5771 5779 5993 ///
	5994 8607 9645 9646 9647

	foreach rn_y of local rn_icd_4 {
		replace renal_ccc = 1 if substr(`pc'`i',1,4)=="`rn_y'"
		}

* GI CCC ICD-9

local gi_icd_3 253 254 504 526 527
local gi_icd_4 4210 4211 4242 4281 4311 4319 4391 4399 4412 4432 4438 ///
	4439 4563 4581 4582 4583 4613 4622 4623 4632 4640 4641 4643 4697 ///
	5051 5059 5280 5282 5283 5284 5285 5286 5471 9624 9636 9702



	foreach gi_x of local gi_icd_3 {
		replace gi_ccc = 1 if substr(`pc'`i',1,3)=="`gi_x'"
		}
	foreach gi_y of local gi_icd_4 {
```

```
                replace gi_ccc = 1 if substr(`pc'`i',1,4)=="`gi_y'"
                }

* HEME/IMMUNO CCC ICD-9

local hi_icd_3 415
local hi_icd_4 4100 4101 4102 4103 4104 4105 4106 4107 4108 4109 4194

        foreach hi_x of local hi_icd_3 {
                replace hemato_immu_ccc = 1 if substr(`pc'`i',1,3)=="`hi_x'"
                }
        foreach hi_y of local hi_icd_4 {
                replace hemato_immu_ccc = 1 if substr(`pc'`i',1,4)=="`hi_y'"
                }

* METABOLIC CCC ICD-9

local mb_icd_3 064 073 624 645
local mb_icd_4 0652 0681 0764 0765 0768 0769 6241 6551 6553 6561 ///
        6563 6841 6849 6851 6859 6861 6869 6871 6879 8606

        foreach mb_x of local mb_icd_3 {
                replace metabolic_ccc = 1 if substr(`pc'`i',1,3)=="`mb_x'"
                }
        foreach mb_y of local mb_icd_4 {
                replace metabolic_ccc = 1 if substr(`pc'`i',1,4)=="`mb_y'"
                }

* CONGENITAL/GENETIC CCC ICD-9

* None

* MALIGNANCY CCC ICD-9

local mg_icd_4 0010 9925

        foreach mg_x of local mg_icd_4 {
```

```
        replace malignancy_ccc = 1 if substr(`pc'`i',1,4)=="`mg_x'"
        }

* NEONATAL CCC ICD-9

* None

* TECHNOLOGY DEPENDENT CCC ICD-9

local td_icd_4 0221 0222 0231 0232 0233 0234 0235 0239 0241 0242 0293 0371 ///
    0372 0379 0393 0397 0492 0050 0051 0053 0054 0055 0057 1751 1752 ///
    3741 3752 3753 3754 3755 3760 3761 3763 3765 3766 3767 3768 3771 ///
    3772 3774 3776 3779 3780 3781 3782 3783 3785 3786 3787 3789 3794 ///
    3795 3796 3797 3798 3981 3982 3983 3984 3985 8945 8946 8947 8948 ///
    8949 3121 3129 3141 3174 3321 3485 9655 9723 3895 3927 3942 3993 ///
    3994 3995 5498 5502 5503 5504 5512 5593 5594 5597 5651 5652 5661 ///
    5662 5672 5673 5674 5675 5721 5722 5993 5994 8607 9645 9646 9647 ///
    4210 4211 4281 4311 4319 4412 4432 4438 4439 4613 4622 4623 4632 ///
    4640 4641 4643 9624 9636 9702 8100 8101 8102 8103 8104 8105 8106 ///
    8107 8108 8109 8130 8131 8132 8133 8134 8135 8136 8137 8138 8139 ///
    8451

    foreach td_x of local td_icd_4 {
        replace tech_dep_ccc = 1 if substr(`pc'`i',1,4)=="`td_x'"
        }

* TRANSPLANT CCC ICD-9

local tx_icd_3 336
local tx_icd_4 3751 3350 3351 3352 5561 5569 4697 5051 5059 5280 5282 ///
    5283 5284 5285 5286 4100 4101 4102 4103 4104 4105 4106 4107 4108 ///
    4109 4194 0091 0092 0093

    foreach tx_w of local tx_icd_3 {
        replace transplant_ccc = 1 if substr(`pc'`i',1,3)=="`tx_w'"
        }
```

```
        foreach tx_x of local tx_icd_4 {
                replace transplant_ccc = 1 if substr(`pc'`i',1,4)=="`tx_x'"
                }
}
}
}
if `icdv'==10 {

* ANALYZE ICD-10 DIAGNOSTIC CODES

gsort -dx_count

forvalues i=1/`n_dxs' {

if `dx'`i' !="" {

* NEUROMUSCULAR CCC ICD-10

local nm_icd_3 F71 F72 F73 G71 G72 G80 G94 Q00 Q01 Q02 Q03 Q04 Q05 ///
        Q06 Q07 G10 G20
local nm_icd_4 E750 E751 E752 E754 F842 G111 G112 G114 G118 G119 G120 ///
        G121 G122 G128 G129 G318 G901 G938 G939 G911 G319 G253 G909 G371 ///
        G372 G378 G835 G839 G931 9782 G935 G210 G218 G230 G231 G232 G238 ///
        G248 G253 G254 G255 G259 G803 R403 Q851 Z982
local nm_icd_5 G3101 G3109 G3189 G9519 G9589 G8190 G8290 G8250 G8251 ///
        G8252 G8253 G8254 I6330 I6350 G2111 G2119 G2402 G2581 G2582 G2583 ///
        G2589 G9782 Z4541 Z4542
local nm_icd_6 G40311 G40301 G40211 G40219 G40411 G40419 G40111 G40119 ///
        G40804 G40911 G40919
local nm_icd_7 T8509XA T85190A T85192A T85199A T8579XA

        foreach nm_v of local nm_icd_3 {
                replace neuromusc_ccc = 1 if substr(`dx'`i',1,3)=="`nm_v'"
                }
        foreach nm_w of local nm_icd_4 {
                replace neuromusc_ccc = 1 if substr(`dx'`i',1,4)=="`nm_w'"
                }
```

```
        foreach nm_x of local nm_icd_5 {
                replace neuromusc_ccc = 1 if substr(`dx'`i',1,5)=="`nm_x'"
                }
        foreach nm_y of local nm_icd_6 {
                replace neuromusc_ccc = 1 if substr(`dx'`i',1,6)=="`nm_y'"
                }
        foreach nm_z of local nm_icd_7 {
                replace neuromusc_ccc = 1 if substr(`dx'`i',1,7)=="`nm_z'"
                }

* CARDIOVASCULAR CCC ICD-10

local cv_icd_3 I42 I43 I44 I45 I47 I48 Q20 Q22 Q23 Q24 Q26
local cv_icd_4 I490 I491 I493 I494 I495 I498 I499 I515 R001 Q212 ///
        Q213 Q214 Q218 Q219 Q251 Q252 Q253 Q254 Q255 Q256 Q257 Q258 ///
        Q259 Q282 Q283 Q289 I340 I348 I360 I368 I370 I378 I509 I517 ///
        Z951 Z941 Z950 Z952 Z953 Z959 I270 I271 I272 I279
local cv_icd_5 I2781 I2789 I5181 Z4502 Z4509
local cv_icd_6 I63139 I63239 Z95810 Z95811 Z95812 Z95818 Z45010 ///
        Z45018
local cv_icd_7 T82519A T82529A T82539A T82599A T82110A T82111A T82120A ///
        T82190A T82191A T8201XA T8202XA T8203XA T8209XA T82211A T82212A ///
        T82213A T82218A T82221A T82222A T82223A T82228A T82518A T82528A ///
        T82538A T82598A T826XXA T827XXA

        foreach cv_v of local cv_icd_3 {
                replace cvd_ccc = 1 if substr(`dx'`i',1,3)=="`cv_v'"
                }
        foreach cv_w of local cv_icd_4 {
                replace cvd_ccc = 1 if substr(`dx'`i',1,4)=="`cv_w'"
                }
        foreach cv_x of local cv_icd_5 {
                replace cvd_ccc = 1 if substr(`dx'`i',1,5)=="`cv_x'"
                }
        foreach cv_y of local cv_icd_6 {
                replace cvd_ccc = 1 if substr(`dx'`i',1,6)=="`cv_y'"
```

```
        }
    foreach cv_z of local cv_icd_7 {
        replace cvd_ccc = 1 if substr(`dx'`i',1,7)=="`cv_z'"
        }

* RESPIRATORY CCC ICD-10

local rp_icd_3 E84 I43 Q30 Q31 Q32 Q33 Q34
local rp_icd_4 P280 Z902 Z430 Z930 Z942 Z990
local rp_icd_5 G4735 I2782 J9500 J9501 J9502 J9503 J9504 J9509 J9620 ///
    Z9911 Z9912
local rp_icd_6 J84112 J95850 T86810 T86811 T86819

    foreach rp_w of local rp_icd_3 {
        replace respiratory_ccc = 1 if substr(`dx'`i',1,3)=="`rp_w'"
        }
    foreach rp_x of local rp_icd_4 {
        replace respiratory_ccc = 1 if substr(`dx'`i',1,4)=="`rp_x'"
        }
    foreach rp_y of local rp_icd_5 {
        replace respiratory_ccc = 1 if substr(`dx'`i',1,5)=="`rp_y'"
        }
    foreach rp_z of local rp_icd_6 {
        replace respiratory_ccc = 1 if substr(`dx'`i',1,6)=="`rp_z'"
        }

* RENAL CCC ICD-10

local rn_icd_3 N18 Q60 Q61 Q62 Q63 Q64
local rn_icd_4 Z905 Z906 G834 N312 N319 Z940 Z936 Z992 Z435 Z436 Z446
local rn_icd_5 Z9350 Z9351 Z9352 Z9359 Z9115 Z4901 Z4902 Z4931 ///
    Z4932 T8610 T8611 T8612
local rn_icd_7 T8571XA

    foreach rn_w of local rn_icd_3 {
        replace renal_ccc = 1 if substr(`dx'`i',1,3)=="`rn_w'"
        }
```

```
        foreach rn_x of local rn_icd_4 {
                replace renal_ccc = 1 if substr(`dx'`i',1,4)=="`rn_x'"
                }
        foreach rn_y of local rn_icd_5 {
                replace renal_ccc = 1 if substr(`dx'`i',1,5)=="`rn_y'"
                }
        foreach rn_z of local rn_icd_7 {
                replace renal_ccc = 1 if substr(`dx'`i',1,7)=="`rn_z'"
                }

* GI CCC ICD-10

local gi_icd_3 K50 K51 K73 K74 Q41 Q42 Q43 Q44 Q45
local gi_icd_4 K754 K760 K761 K762 K763 K768 Q390 Q391 Q392 Q393 Q394 ///
        I820 K551 K562 K593 Z980 Z903 Z944 Z931 Z932 Z933 Z934 Z431 Z432 ///
        Z433 Z434
local gi_icd_5 Z9049 K9420 K9422 K9423 K9429 Z9482 Z9483 Z4651 Z4659 ///
        T8640 T8641 T8642
local gi_icd_6 T86890 T86891 T86899 T86850 T86851 T86859

        foreach gi_w of local gi_icd_3 {
                replace gi_ccc = 1 if substr(`dx'`i',1,3)=="`gi_w'"
                }
        foreach gi_x of local gi_icd_4 {
                replace gi_ccc = 1 if substr(`dx'`i',1,4)=="`gi_x'"
                }
        foreach gi_y of local gi_icd_5 {
                replace gi_ccc = 1 if substr(`dx'`i',1,5)=="`gi_y'"
                }
        foreach gi_z of local gi_icd_6 {
                replace gi_ccc = 1 if substr(`dx'`i',1,7)=="`gi_z'"
                }

* HEME/IMMUNO CCC ICD-10

local hi_icd_3 B20 D55 D56 D57 D58 D60 D61 D71 D720 D80 D81 D82 ///
```

```
	D83 D84 D85 D87 D88 D86 B21 B22 B23 B24 D66 Z21
local hi_icd_4 M303 M359 D700 D704 D682 D761 D762 D763 D869 M300 ///
	M310 M311 M314 M316 M340 M341 M349
local hi_icd_5 D6941 D6942 M3130 M3210 M3390

	foreach hi_w of local hi_icd_3 {
		replace hemato_immu_ccc = 1 if substr(`dx'`i',1,3)=="`hi_w'"
		}
	foreach hi_x of local hi_icd_4 {
		replace hemato_immu_ccc = 1 if substr(`dx'`i',1,4)=="`hi_x'"
		}
	foreach hi_y of local hi_icd_5 {
		replace hemato_immu_ccc = 1 if substr(`dx'`i',1,5)=="`hi_y'"
		}

* METABOLIC CCC ICD-10

local mb_icd_3 E75 E85 E88
local mb_icd_4 D841 E700 E702 E703 E704 E705 E708 E710 E711 E712 E713 ///
	E714 E715 E720 E721 E722 E723 E724 E728 E729 E740 E741 E742 E743 ///
	E744 E748 E749 E760 E761 E762 E763 E770 E771 E780 E781 E782 E783 ///
	E784 E785 E786 E787 E788 E789 E791 E798 E804 E805 E806 E807 E830 ///
	E831 E833 E834 H498 E009 E230 E232 E222 E233 E237 E240 E242 E243 ///
	E248 E249 E250 E258 E259 Z794
local mb_icd_5 E2681 Z4681 Z9641

	foreach mb_w of local mb_icd_3 {
		replace metabolic_ccc = 1 if substr(`dx'`i',1,3)=="`mb_w'"
		}
	foreach mb_x of local mb_icd_4 {
		replace metabolic_ccc = 1 if substr(`dx'`i',1,4)=="`mb_x'"
		}
	foreach mb_y of local mb_icd_5 {
		replace metabolic_ccc = 1 if substr(`dx'`i',1,5)=="`mb_y'"
		}

* CONGENITAL/GENETIC CCC ICD-10
```

```
local cg_icd_3 Q77 Q93 Q97 Q98 Q81

local cg_icd_4 E343 K449 M410 M412 M418 M419 M965 Q722 Q750 Q752 ///

       Q759 Q760 Q761 Q762 Q764 Q765 Q766 Q767 Q780 Q781 Q782 Q783 ///

       Q784 Q788 Q789 Q790 Q791 Q792 Q793 Q794 Q799 Q795 Q897 Q899 ///

       Q909 Q913 Q914 Q917 Q928 Q950 Q969 Q992 Q998 Q999 Q898

local cg_icd_5 M4130 M4330 Q8740 Q8781 Q8789


       foreach cg_x of local cg_icd_3 {

              replace congeni_genetic_ccc = 1 if substr(`dx'`i',1,3)=="`cg_x'"

              }

       foreach cg_y of local cg_icd_4 {

              replace congeni_genetic_ccc = 1 if substr(`dx'`i',1,4)=="`cg_y'"

              }

       foreach cg_z of local cg_icd_5 {

              replace congeni_genetic_ccc = 1 if substr(`dx'`i',1,5)=="`cg_z'"

              }

* MALIGNANCY CCC ICD-10


local mg_icd_1 C

local mg_icd_3 D00 D01 D02 D03 D04 D05 D06 D07 D08 D09 D37 D38 D39 D40 ///

       D41 D42 D43 D44 D45 D46 D47 D48 D49

local mg_icd_4 D3A0 Q850

local mg_icd_5 Z9481 Z9484 T8600 T8601 T8602 T8609


       foreach mg_w of local mg_icd_1 {

              replace malignancy_ccc = 1 if substr(`dx'`i',1,1)=="`mg_w'"

              }

       foreach mg_x of local mg_icd_3 {

              replace malignancy_ccc = 1 if substr(`dx'`i',1,3)=="`mg_x'"

              }

       foreach mg_y of local mg_icd_4 {

              replace malignancy_ccc = i if substr(`dx'`i',1,4)=="`mg_y'"

              }

       foreach mg_z of local mg_icd_5 {

              replace malignancy_ccc = 1 if substr(`dx'`i',1,5)=="`mg_z'"
```

```
		}

* NEONATAL CCC ICD-10

local nn_icd_3 P84
local nn_icd_4 P052 P059 P100 P101 P104 P524 P528 P115 P916 P210 P250 ///
	P251 P253 P258 P219 P270 P271 P278 P350 P351 P560 P570 P578 ///
	P613 P614 P773 P832 P912
local nn_icd_5 P0501 P0511 P0502 P0512 P0701 P0702 P0721 P0722 P0723 ///
	P0724 P0725 P2521 P2522

	foreach nn_x of local nn_icd_3 {
		replace neonatal_ccc = 1 if substr(`dx'`i',1,3)=="`nn_x'"
		}
	foreach nn_y of local nn_icd_4 {
		replace neonatal_ccc = 1 if substr(`dx'`i',1,4)=="`nn_y'"
		}
	foreach nn_z of local nn_icd_5 {
		replace neonatal_ccc = 1 if substr(`dx'`i',1,5)=="`nn_z'"
		}

* TECHNOLOGY CCC ICD-10

local td_icd_4 Z940 Z982 Z950 Z952 Z953 Z959 Z430 Z930 Z942 Z990 Z936 ///
	Z992 Z435 Z436 Z446 Z931 Z932 Z933 Z934 Z431 Z432 Z433 Z434 T865 ///
	T872 Y831 Y833
local td_icd_5 Z4502 Z4509 J9500 J9501 J9502 J9503 J9504 J9509 Z9911 ///
	Z9912 Z9350 Z9351 Z9352 Z9359 Z9115 K9420 K9422 K9423 K9429 ///
	Z4651 Z4659 Z4681 Z9641 T8690 T8691 T8692 T8699 T8610 T8611 ///
	T8612 T8640 T8641 T8642 T8620 T8621 T8622 T8600 T8601 T8602 ///
	T8609
local td_icd_6 Z45441 Z45442 Z95810 Z95811 Z95812 Z95818 Z45010 Z45018 ///
	J95850 T86810 T86811 T86819 T86890 T86891 T86899 T86850 T86851 ///
	T86859 T86890 T86891 T86899 T870X9 T871X9
local td_icd_7 T8509XA T85190A T85192A T85199A T8579XA T82519A ///
	T82529A T82539A T82599A T82110A T82111A T82120A T82121A T82190A T82191A ///
	T8201XA T8202XA T8203XA T8209XA T82211A T82212A T82213A T82218A T82221A ///
```

```
        T82222A T82223A T82228A T82518A T82528A T82538A T82598A T826XXA T827XXA ///
        T8571XA T84019A T84029A T84039A T84049A T84059A T84069A T84099A T84498A ///
        T84119A T84129A T84199A T84498A T8450XA T8460XA T847XXA

        foreach td_w of local td_icd_4 {
                replace tech_dep_ccc = 1 if substr(`dx'`i',1,4)=="`td_w'"
                }
        foreach td_x of local td_icd_5 {
                replace tech_dep_ccc = 1 if substr(`dx'`i',1,5)=="`td_x'"
                }
        foreach td_y of local td_icd_6 {
                replace tech_dep_ccc = 1 if substr(`dx'`i',1,6)=="`td_y'"
                }
        foreach td_z of local td_icd_7 {
                replace tech_dep_ccc = 1 if substr(`dx'`i',1,7)=="`td_z'"
                }

* TRANSPLANTATION CCC ICD-10

local tx_icd_4 T865
local tx_icd_5 T8600 T8601 T8602 T8609 T8610 T8611 T8612 T8620 T8621 T8622 ///
        T8640 T8641 T8642 T8690 T8691 T8692 T8699
local tx_icd_6 T86810 T86811 T86819 T86890 T86891 T86899 T86850 ///
        T86851 T86859 T86890 T86891 T86899

        foreach tx_x of local tx_icd_4 {
                replace transplant_ccc = 1 if substr(`dx'`i',1,4)=="`tx_x'"
                }
        foreach tx_y of local tx_icd_5 {
                replace transplant_ccc = 1 if substr(`dx'`i',1,5)=="`tx_y'"
                }
        foreach tx_z of local tx_icd_6 {
                replace transplant_ccc = 1 if substr(`dx'`i',1,6)=="`tx_z'"
                }
}
}
```

```
* ANALYZE ICD-10 PROCEDURE CODES

gsort -pc_count

forvalues i=1/`n_pcs' {

if `pc'`i' !="" {

* NEUROMUSCULAR CCC ICD-10

local nm_icd_7 0016070 0016071 0016072 0016073 0016074 0016075 0016076 0016077 ///
       0016078 001607B 00160J0 00160J1 00160J2 00160J4 00160J5 00160J6 00160J7 ///
       00160J8 00160JB 00160K0 00160K1 00160K2 00160K3 00160K4 00160K5 00160K6 ///
       00160K7 00160K8 00160KB 0016370 0016371 0016372 0016373 0016374 0016375 ///
       0016376 0016377 0016378 001637B 00163J0 00163J1 00163J2 00163J3 00163J4 ///
       00163J5 00163J6 00163J7 00163J8 00163JB 00163K0 00163K1 00163K2 00163K3 ///
       00163K4 00163K5 00163K6 00163K7 00163K8 00163KB 001U074 001U076 001U077 ///
       001U079 001U0J4 001U0J6 001U0J7 001U0J9 001U0K4 001U0K6 001U0K7 001U0K9 ///
       001U374 001U376 001U377 001U379 001U3J4 001U3J6 001U3J7 001U3J9 001U3K4 ///
       001U3K6 001U3K7 001U3K9 009600Z 009630Z 009640Z 00B70ZZ 00B73ZZ 00B74ZZ ///
       00H00MZ 00H03MZ 00H04MZ 00H60MZ 00H63MZ 00H64MZ 00HE0MZ 00HE3MZ 00HE4MZ ///
       00HU0MZ 00HU3MZ 00HU4MZ 00HV0MZ 00HV3MZ 00HV4MZ 00T70ZZ 00T73ZZ 00T74ZZ ///
       00W60JZ 00W63JZ 00W64JZ 00WU0JZ 00WU3JZ 01HY0MZ 01HY3MZ 0DH60MZ 0DH63MZ ///
       0DH64MZ 0W110J9 0W110JB 0W110JG 0W110JJ 3E1Q38X 3E1Q38Z

       foreach nm_z of local nm_icd_7 {
              replace neuromusc_ccc = 1 if substr(`pc'`i',1,7)=="`nm_z'"
              }

* CARDIOVASCULAR CCC ICD-10

local cv_icd_7 02170ZP 02170ZQ 02170ZR 02BK0ZZ 02H40JZ 02H40KZ 02H43JZ 02H44JZ ///
       02H44KZ 02H60JZ 02H60KZ 02H63JZ 02H63KZ 02H63MZ 02H64JZ 02H64KZ 02H70KZ ///
       02H73JZ 02H73KZ 02H73MZ 02H74KZ 02HA0QZ 02HA0RS 02HA0RZ 02HA3QZ 02HA3RS ///
       02HA4QZ 02HA4RS 02HK0JZ 02HK0KZ 02HK3JZ 02HK3KZ 02HK3MZ 02HK4JZ 02HK4KZ ///
       02HL0JZ 02HL0KZ 02HL0MZ 02HL3JZ 02HL3KZ 02HL3MZ 02HL4JZ 02HL4KZ 02HL4MZ ///
```

```
	02HN0JZ 02HN0KZ 02HN0MZ 02HN3JZ 02HN3KZ 02HN3MZ 02HN4JZ 02HN4KZ 02HN4MZ ///
	02LR0ZT 02LS0ZZ 02LT0ZZ 02NH0ZZ 02RK0JZ 02RL0JZ 02RM0JZ 02RP0JZ 02RQ07Z ///
	02RQ0JZ 02RR07Z 02RR0JZ 02SP0ZZ 02SW0ZZ 02U70JZ 02UA0JZ 02UA3JZ 02UA4JZ ///
	02VR0ZT 02WA0JZ 02WA0QZ 02WA0RZ 02WA3QZ 02WA3RZ 02WA4QZ 02WA4RZ 02YA0Z0 ///
	02YA0Z1 02YA0Z2 03HK0MZ 03HK3MZ 03HK4MZ 03HL0MZ 03HL3MZ 03HL4MZ 03WY0MZ ///
	03WY3MZ 03WY4MZ 0JH600Z 0JH605Z 0JH606Z 0JH607Z 0JH608Z 0JH609Z 0JH60AZ ///
	0JH60MZ 0JH60PZ 0JH630Z 0JH635Z 0JH636Z 0JH637Z 0JH638Z 0JH639Z 0JH63AZ ///
	0JH63MZ 0JH63PZ 0JH70MZ 0JH73MZ 0JH800Z 0JH805Z 0JH806Z 0JH807Z 0JH808Z ///
	0JH809Z 0JH80AZ 0JH80MZ 0JH80PZ 0JH830Z 0JH835Z 0JH836Z 0JH837Z 0JH838Z ///
	0JH839Z 0JH83AZ 0JH83MZ 0JH83PZ 0JWT0MZ 0JWT0PZ 0JWT3MZ 0JWT3PZ 0JWTXMZ ///
	4B02XSZ 4B02XTZ 5A02110 5A02116 5A0211D 5A02210 5A02216 5A0221D

	foreach cv_z of local cv_icd_7 {
		replace cvd_ccc = 1 if substr(`pc'`i',1,7)=="`cv_z'"
		}

* RESPIRATORY CCC ICD-10

local rp_icd_7 0B110F4 0B110Z4 0B113F4 0B113Z4 0B114F4 0B114Z4 0B21XFZ 0BHR0MZ ///
	0BHR3MZ 0BHR4MZ 0BHS0MZ 0BHS3MZ 0BHS4MZ 0BTC0ZZ 0BTC4ZZ 0BTD0ZZ 0BTD4ZZ ///
	0BTF0ZZ 0BTF4ZZ 0BTG0ZZ 0BTG4ZZ 0BTJ0ZZ 0BTJ4ZZ 0BTK0ZZ 0BTK4ZZ 0BTL0ZZ ///
	0BTL4ZZ 0BTM0ZZ 0BTM4ZZ 0BW10FZ 0BW13FZ 0BW14FZ 0BYC0Z0 0BYC0Z1 0BYC0Z2 ///
	0BYD0Z0 0BYD0Z1 0BYD0Z2 0BYF0Z0 0BYF0Z1 0BYF0Z2 0BYG0Z0 0BYG0Z1 0BYG0Z2 ///
	0BYH0Z0 0BYH0Z1 0BYH0Z2 0BYJ0Z0 0BYJ0Z1 0BYJ0Z2 0BYK0Z0 0BYK0Z1 0BYK0Z2 ///
	0BYL0Z0 0BYL0Z1 0BYL0Z2 0BYM0Z0 0BYM0Z1 0BYM0Z2 0CTS0ZZ 0CTS4ZZ 0CTS7ZZ ///
	0CTS8ZZ 0JH604Z 0JH634Z 0JH804Z 0JH834Z 0WQ6XZ2 3E1F78Z

	foreach rp_y of local rp_icd_7 {
		replace respiratory_ccc = 1 if substr(`pc'`i',1,7)=="`rp_y'"
		}

* RENAL CCC ICD-10

local rn_icd_7 031209D 031209F 03120AD 03120AF 03120JD 03120JF 03120KD 03120KF ///
	03120ZD 03120ZF 031309D 031309F 03130AD 03130AF 03130JD 03130JF 03130KD ///
	03130KF 03130ZD 03130ZF 031409D 031409F 03140AD 03140AF 03140JD 03140JF ///
```

```
	03140KD 03140KF 03140ZD 03140ZF 031509D 031509F 03150AD 03150AF 03150JF ///
	03150KF 03150ZF 031609F 03160AD 03160AF 03160JD 03160KD 03160KF 03160ZD ///
	03160ZF 031709D 031709F 03170AD 03170AF 03170JD 03170JF 03170KD 03170KF ///
	03170ZD 03170ZF 031809D 031809F 03180AD 03180AF 03180JD 03180JF 03180KD ///
	03180KF 03180ZD 03180ZF 031909F 03190AF 03190JF 03190KF 03190ZF 031A09F ///
	031A0AF 031A0JF 031A0KF 031A0ZF 031B09F 031B0AF 031B0KF 031B0ZF 031C09F ///
	031C0AF 031C0JF 031C0KF 031C0ZF 03WY0JZ 03WY3JZ 03WY4JZ 03WYXJZ 05HY33Z ///
	0JH60WZ 0JH60XZ 0JH63WZ 0JH63XZ 0JH80WZ 0JH80XZ 0JH83WZ 0JH83XZ 0JHD0XZ ///
	0JHD3WZ 0JHD3XZ 0JHF0WZ 0JHF0XZ 0JHF3WZ 0JHF3XZ 0JHL0WZ 0JHL0XZ 0JHL3WZ ///
	0JHL3XZ 0JHM0WZ 0JHM0XZ 0JHM3WZ 0JHM3XZ 0T130ZB 0T134ZB 0T140ZB 0T144ZB ///
	0T16079 0T1607C 0T160J9 0T160JC 0T160K9 0T160KC 0T160KD 0T160Z8 0T160Z9 ///
	0T160ZA 0T160ZC 0T160ZD 0T163JD 0T16479 0T1647D 0T164J9 0T164JC 0T164JD ///
	0T164K9 0T164KC 0T164KD 0T164Z8 0T164Z9 0T164ZA 0T164ZC 0T164ZD 0T17079 ///
	0T1707C 0T1707D 0T170J9 0T170JC 0T170JD 0T170K9 0T170KD 0T170Z8 0T170Z9 ///
	0T170ZA 0T170ZC 0T170ZD 0T173JD 0T1747C 0T1747D 0T174J9 0T174JC 0T174JD ///
	0T174K9 0T174KC 0T174KD 0T174Z8 0T174Z9 0T174ZA 0T174ZC 0T174ZD 0T18079 ///
	0T1807C 0T1807D 0T180J9 0T180JC 0T180JD 0T180K9 0T180KC 0T180KD 0T180Z8 ///
	0T180Z9 0T180ZA 0T180ZC 0T180ZD 0T18479 0T1847C 0T1847D 0T184J9 0T184JC ///
	0T184JD 0T184K9 0T184KC 0T184KD 0T184Z8 0T184Z9 0T184ZA 0T184ZC 0T184ZD ///
	0T1B0ZD 0T1B4ZD 0T25X0Z 0T29X0Z 0T29XYZ 0T2BX0Z 0T9000Z 0T9030Z 0T9040Z ///
	0T9070Z 0T9080Z 0T9100Z 0T9130Z 0T9140Z 0T9170Z 0T9180Z 0T9370Z 0T9380Z ///
	0T9470Z 0T9480Z 0TB60ZZ 0TB63ZZ 0TB64ZZ 0TB67ZZ 0TB68ZZ 0TB70ZZ 0TB74ZZ ///
	0TB77ZZ 0TB78ZZ 0TQ67ZZ 0TQ77ZZ 0TT00ZZ 0TT04ZZ 0TT10ZZ 0TT14ZZ 0TT20ZZ ///
	0TT24ZZ 0TT60ZZ 0TT64ZZ 0TT67ZZ 0TT68ZZ 0TT70ZZ 0TT74ZZ 0TT77ZZ 0TT78ZZ ///
	0TTB0ZZ 0TTB4ZZ 0TTB7ZZ 0TTB8ZZ 0TTD0ZZ 0TTD4ZZ 0TTD7ZZ 0TTD8ZZ 0TY00Z0 ///
	0TY00Z1 0TY00Z2 0TY10Z0 0TY10Z1 0TY10Z2 3E1K38Z 3E1M39Z 5A1D60Z

	foreach rn_z of local rn_icd_7 {
		replace renal_ccc = 1 if substr(`pc'`i',1,7)=="`rn_z'"
		}


* GI CCC ICD-10

local gi_icd_7 0CT70ZZ 0CT7XZZ 0D11074 0D110J4 0D110K4 0D110Z4 0D113J4 0D11474 ///
	0D114J4 0D114K4 0D114Z4 0D13079 0D1307A 0D1307B 0D15074 0D150J4 0D150K4 ///
	0D150Z4 0D153J4 0D15474 0D154J4 0D154K4 0D154Z4 0D16074 0D1607A 0D160J4 ///
```

```
        0D160J9 0D160JA 0D160K4 0D160K9 0D160KA 0D160Z4 0D160ZA 0D163J4 0D16474 ///
        0D164J4 0D164J9 0D164JA 0D164K4 0D164K9 0D164KA 0D164Z4 0D16874 0D168J4 ///
        0D168J9 0D168JA 0D168K4 0D168K9 0D168KA 0D168Z4 0D1B0Z4 0D1B4Z4 0D1B8Z4 ///
        0D1H0Z4 0D1H4Z4 0D1H8Z4 0D1K0Z4 0D1K4Z4 0D1K8Z4 0D1L0Z4 0D1L4Z4 0D1L8Z4 ///
        0D1N0Z4 0D1N4Z4 0D1N8Z4 0D20X0Z 0D20XUZ 0D20XYZ 0D787ZZ 0D7E7ZZ 0DBB7ZZ ///
        0DH50DZ 0DH50UZ 0DH53DZ 0DH53UZ 0DH54DZ 0DH54UZ 0DH57DZ 0DH57UZ 0DH58DZ ///
        0DH58UZ 0DH63UZ 0DH64UZ 0DHA3UZ 0DHA4UZ 0DHA8UZ 0DN87ZZ 0DNE7ZZ 0DT50ZZ ///
        0DT54ZZ 0DT57ZZ 0DT58ZZ 0DT60ZZ 0DT64ZZ 0DT67ZZ 0DT68ZZ 0DT80ZZ 0DT84ZZ ///
        0DT87ZZ 0DT88ZZ 0DT90ZZ 0DT94ZZ 0DT97ZZ 0DT98ZZ 0DTE0ZZ 0DTE4ZZ 0DTE7ZZ ///
        0DTE8ZZ 0DW04UZ 0DW08UZ 0DY80Z0 0DY80Z1 0DY80Z2 0DYE0Z0 0DYE0Z1 0DYE0Z2 ///
        0FT00ZZ 0FT04ZZ 0FTG0ZZ 0FTG4ZZ 0FY00Z0 0FY00Z1 0FY00Z2 0FYG0Z0 0FYG0Z1 ///
        0FYG0Z2 0WQFXZ2 3E030U0 3E030U1 3E033U0 3E033U1 3E0J3U0 3E0J3U1 3E0J7U0 ///
        3E0J7U1 3E0J8U0 3E0J8U1 3E1G78Z 3E1H78Z

        foreach gi_z of local gi_icd_7 {
                replace gi_ccc = 1 if substr(`pc'`i',1,7)=="`gi_z'"
                }

* HEME/IMMUNO CCC ICD-10

local hi_icd_7 07TP0ZZ 07TP4ZZ 07YP0Z0 07YP0Z1 07YP0Z2 30230AZ 30230G0 30230G1 ///
        30230X0 30230X1 30230Y0 30230Y1 30233AZ 30233G0 30233G1 30233X0 30233X1 ///
        30233Y0 30233Y1 30240AZ 30240G0 30240G1 30240X0 30240X1 30240Y0 30240Y1 ///
        30243AZ 30243G0 30243G1 30243X0 30243X1 30243Y0 30243Y1 30250G0 30250G1 ///
        30250X0 30250X1 30250Y0 30250Y1 30253G0 30253G1 30253X0 30253X1 30253Y0 ///
        30253Y1 30260G0 30260G1 30260X0 30260X1 30260Y0 30260Y1 30263G0 30263G1 ///
        30263X0 30263X1 30263Y0 30263Y1

        foreach hi_z of local hi_icd_7 {
                replace hemato_immu_ccc = 1 if substr(`pc'`i',1,7)=="`hi_z'"
                }

* METABOLIC CCC ICD-10

local mb_icd_7 0GT00ZZ 0GT04ZZ 0GT40ZZ 0GT44ZZ 0GTK0ZZ 0GTK4ZZ 0GTR0ZZ 0GTR4ZZ ///
        0JH60VZ 0JH63VZ 0JH70VZ 0JH73VZ 0JH80VZ 0JH83VZ 0JHD0VZ 0JHD3VZ 0JHF0VZ ///
```

```
        0JHF3VZ 0JHG0VZ 0JHG3VZ 0JHH0VZ 0JHH3VZ 0JHL0VZ 0JHL3VZ 0JHM0VZ 0JHM3VZ ///
        0JHN0VZ 0JHN3VZ 0JHP0VZ 0JHP3VZ 0JHT0VZ 0JHT3VZ 0UT20ZZ 0UT24ZZ 0UT27ZZ ///
        0UT28ZZ 0UT2FZZ 0UT40ZZ 0UT44ZZ 0UT47ZZ 0UT48ZZ 0UT70ZZ 0UT74ZZ 0UT90ZZ ///
        0UT94ZZ 0UT97ZZ 0UT98ZZ 0UT9FZZ 0UTC0ZZ 0UTC7ZZ 0UTC8ZZ 0VTC0ZZ 0VTC4ZZ ///
        0W4M070 0W4M0J0 0W4M0K0 0W4M0Z0 0W4N071 0W4N0J1 0W4N0K1 0W4N0Z1

        foreach mb_z of local mb_icd_7 {
                replace metabolic_ccc = 1 if substr(`pc'`i',1,7)=="`mb_z'"
                }

* CONGENITAL/GENETIC CCC ICD-10

* None

* MALIGNANCY CCC ICD-10

local mg_icd_7 3E00X05 3E01305 3E02305 3E03005 3E03305 3E04005 3E04305 3E05005 ///
        3E05305 3E06005 3E06305 3E0A305 3E0F305 3E0F705 3E0F805 3E0G305 3E0G705 ///
        3E0G805 3E0H305 3E0H705 3E0H805 3E0J305 3E0J705 3E0J805 3E0K305 3E0K705 ///
        3E0K805 3E0L305 3E0L705 3E0M305 3E0M705 3E0N305 3E0N705 3E0N805 3E0P305 ///
        3E0P705 3E0P805 3E0Q305 3E0Q705 3E0R305 3E0S305 3E0V305 3E0W305 3E0Y305 ///
        3E0Y705

        foreach mg_z of local mg_icd_7 {
                replace malignancy_ccc = 1 if substr(`pc'`i',1,7)=="`mg_z'"
                }

* NEONATAL CCC ICD-10

* None

* TECHNOLOGY CCC ICD-10

local td_icd_7 00160J0 00160J1 00160J2 00160J4 00160J5 00160J6 00160J7 00160J8 ///
        00160JB 00160K0 00160K1 00160K2 00160K3 00160K4 00160K5 00160K6 00160K7 ///
        00160K8 00160KB 00163J0 00163J1 00163J2 00163J3 00163J4 00163J5 00163J6 ///
        00163J7 00163J8 00163JB 00163K0 00163K1 00163K2 00163K3 00163K4 00163K5 ///
```

00163K6 00163K7 00163K8 00163KB 001U0J4 001U0J6 001U0J7 001U0J9 001U0K4 ///

001U0K6 001U0K7 001U0K9 001U3J4 001U3J6 001U3J7 001U3J9 001U3K4 001U3K6 ///

001U3K7 001U3K9 009600Z 009630Z 009640Z 00H00MZ 00H03MZ 00H04MZ 00H60MZ ///

00H63MZ 00H64MZ 00HE0MZ 00HE3MZ 00HE4MZ 00HU0MZ 00HU3MZ 00HU4MZ 00HV0MZ ///

00HV3MZ 00HV4MZ 00W60JZ 00W63JZ 00W64JZ 00WU0JZ 00WU3JZ 01HY0MZ 01HY3MZ ///

0DH60MZ 0DH63MZ 0DH64MZ 0W110J9 0W110JB 0W110JG 0W110JJ 3E1Q38X 3E1Q38Z ///

02H40JZ 02H40KZ 02H43JZ 02H44JZ 02H44KZ 02H60JZ 02H60KZ 02H63JZ 02H63KZ ///

02H63MZ 02H64JZ 02H64KZ 02H70KZ 02H73JZ 02H73KZ 02H73MZ 02H74KZ 02HA0QZ ///

02HA0RS 02HA0RZ 02HA3QZ 02HA3RS 02HA4QZ 02HA4RS 02HK0JZ 02HK0KZ 02HK3JZ ///

02HK3KZ 02HK3MZ 02HK4JZ 02HK4KZ 02HL0JZ 02HL0KZ 02HL0MZ 02HL3JZ 02HL3KZ ///

02HL3MZ 02HL4JZ 02HL4KZ 02HL4MZ 02HN0JZ 02HN0KZ 02HN0MZ 02HN3JZ 02HN3KZ ///

02HN3MZ 02HN4JZ 02HN4KZ 02HN4MZ 02WA0QZ 02WA0RZ 02WA3QZ 02WA3RZ 02WA4QZ ///

02WA4RZ 03HK0MZ 03HK3MZ 03HK4MZ 03HL0MZ 03HL3MZ 03HL4MZ 03WY0MZ 03WY3MZ ///

03WY4MZ 0JH600Z 0JH605Z 0JH606Z 0JH607Z 0JH608Z 0JH609Z 0JH60AZ 0JH60MZ ///

0JH60PZ 0JH630Z 0JH635Z 0JH636Z 0JH637Z 0JH638Z 0JH639Z 0JH63AZ 0JH63MZ ///

0JH63PZ 0JH70MZ 0JH73MZ 0JH800Z 0JH805Z 0JH806Z 0JH807Z 0JH808Z 0JH809Z ///

0JH80AZ 0JH80MZ 0JH80PZ 0JH830Z 0JH835Z 0JH836Z 0JH837Z 0JH838Z 0JH839Z ///

0JH83AZ 0JH83MZ 0JH83PZ 0JWT0MZ 0JWT0PZ 0JWT3MZ 0JWT3PZ 0JWTXMZ 4B02XSZ ///

4B02XTZ 5A02110 5A02116 5A0211D 5A02210 5A02216 5A0221D 0B110F4 0B113F4 ///

0B114F4 0B21XFZ 0BHR0MZ 0BHR3MZ 0BHR4MZ 0BHS0MZ 0BHS3MZ 0BHS4MZ 0BW10FZ ///

0BW13FZ 0BW14FZ 0JH604Z 0JH634Z 0JH804Z 0JH834Z 0WQ6XZ2 3E1F78Z 031209D ///

031209F 03120AD 03120AF 03120JD 03120JF 03120KD 03120KF 03120ZD 03120ZF ///

031309D 031309F 03130AD 03130AF 03130JD 03130JF 03130KD 03130KF 03130ZD ///

03130ZF 031409D 031409F 03140AD 03140AF 03140JD 03140JF 03140KD 03140KF ///

03140ZD 03140ZF 031509D 031509F 03150AD 03150AF 03150JF 03150KF 03150ZF ///

031609F 03160AD 03160AF 03160JD 03160KD 03160KF 03160ZD 03160ZF 031709D ///

031709F 03170AD 03170AF 03170JD 03170JF 03170KD 03170KF 03170ZD 03170ZF ///

031809D 031809F 03180AD 03180AF 03180JD 03180JF 03180KD 03180KF 03180ZD ///

03180ZF 031909F 03190AF 03190JF 03190KF 03190ZF 031A09F 031A0AF 031A0JF ///

031A0KF 031A0ZF 031B09F 031B0AF 031B0KF 031B0ZF 031C09F 031C0AF 031C0JF ///

031C0KF 031C0ZF 03WY0JZ 03WY3JZ 03WY4JZ 03WYXJZ 05HY33Z 0JH60WZ 0JH60XZ ///

0JH63WZ 0JH63XZ 0JH80WZ 0JH80XZ 0JH83WZ 0JH83XZ 0JHD0XZ 0JHD3WZ 0JHD3XZ ///

0JHF0WZ 0JHF0XZ 0JHF3WZ 0JHF3XZ 0JHL0WZ 0JHL0XZ 0JHL3WZ 0JHL3XZ 0JHM0WZ ///

0JHM0XZ 0JHM3WZ 0JHM3XZ 0T130ZB 0T134ZB 0T140ZB 0T144ZB 0T16079 0T1607C ///

0T160J9 0T160JC 0T160K9 0T160KC 0T160KD 0T160Z9 0T160ZC 0T160ZD 0T163JD ///

0T16479 0T1647D 0T164J9 0T164JC 0T164JD 0T164K9 0T164KC 0T164KD 0T164Z9 ///

0T164ZC 0T164ZD 0T17079 0T1707C 0T1707D 0T170J9 0T170JC 0T170JD 0T170K9 ///

0T170KD 0T170Z9 0T170ZC 0T170ZD 0T173JD 0T1747C 0T1747D 0T174J9 0T174JC ///

0T174JD 0T174K9 0T174KC 0T174KD 0T174Z9 0T174ZC 0T174ZD 0T18079 0T1807C ///

0T1807D 0T180J9 0T180JC 0T180JD 0T180K9 0T180KC 0T180KD 0T180Z9 0T180ZC ///

0T180ZD 0T18479 0T1847C 0T1847D 0T184J9 0T184JC 0T184JD 0T184K9 0T184KC ///

0T184KD 0T184Z9 0T184ZC 0T184ZD 0T1B0ZD 0T1B4ZD 0T25X0Z 0T29X0Z 0T29XYZ ///

0T2BX0Z 0T9000Z 0T9030Z 0T9040Z 0T9070Z 0T9080Z 0T9100Z 0T9130Z 0T9140Z ///

0T9170Z 0T9180Z 0T9370Z 0T9380Z 0T9470Z 0T9480Z 0TQ67ZZ 0TQ77ZZ 3E1K38Z ///

3E1M39Z 5A1D60Z 0D11074 0D110J4 0D110K4 0D110Z4 0D113J4 0D11474 0D114J4 ///

0D114K4 0D114Z4 0D15074 0D150J4 0D150K4 0D150Z4 0D153J4 0D15474 0D154J4 ///

0D154K4 0D154Z4 0D16074 0D160J4 0D160J9 0D160JA 0D160K4 0D160K9 0D160KA ///

0D160Z4 0D163J4 0D16474 0D164J4 0D164J9 0D164JA 0D164K4 0D164K9 0D164KA ///

0D164Z4 0D16874 0D168J4 0D168J9 0D168JA 0D168K4 0D168K9 0D168KA 0D168Z4 ///

0D1B0Z4 0D1B4Z4 0D1B8Z4 0D1H0Z4 0D1H4Z4 0D1H8Z4 0D1K0Z4 0D1K4Z4 0D1K8Z4 ///

0D1L0Z4 0D1L4Z4 0D1L8Z4 0D1N0Z4 0D1N4Z4 0D1N8Z4 0D20X0Z 0D20XUZ 0D20XYZ ///

0D787ZZ 0D7E7ZZ 0DBB7ZZ 0DH50DZ 0DH50UZ 0DH53DZ 0DH53UZ 0DH54DZ 0DH54UZ ///

0DH57DZ 0DH57UZ 0DH58DZ 0DH58UZ 0DH63UZ 0DH64UZ 0DHA3UZ 0DHA4UZ 0DHA8UZ ///

0DN87ZZ 0DNE7ZZ 0DW04UZ 0DW08UZ 0WQFXZ2 3E1G78Z 3E1H78Z 0JH60VZ 0JH63VZ ///

0JH70VZ 0JH73VZ 0JH80VZ 0JH83VZ 0JHD0VZ 0JHD3VZ 0JHF0VZ 0JHF3VZ 0JHG0VZ ///

0JHG3VZ 0JHH0VZ 0JHH3VZ 0JHL0VZ 0JHL3VZ 0JHM0VZ 0JHM3VZ 0JHN0VZ 0JHN3VZ ///

0JHP0VZ 0JHP3VZ 0JHT0VZ 0JHT3VZ 0RG00J0 0RG00J1 0RG00JJ 0RG00K0 0RG00K1 ///

0RG00KJ 0RG00Z0 0RG00Z1 0RG00ZJ 0RG03J0 0RG03J1 0RG03JJ 0RG03K0 0RG03K1 ///

0RG03KJ 0RG03Z0 0RG03Z1 0RG03ZJ 0RG04J0 0RG04J1 0RG04JJ 0RG04K0 0RG04K1 ///

0RG04KJ 0RG04Z0 0RG04Z1 0RG04ZJ 0RG10J0 0RG10J1 0RG10JJ 0RG10K0 0RG10K1 ///

0RG10KJ 0RG10Z0 0RG10Z1 0RG10ZJ 0RG13J0 0RG13J1 0RG13JJ 0RG13K0 0RG13K1 ///

0RG13KJ 0RG13Z0 0RG13Z1 0RG13ZJ 0RG14J0 0RG14J1 0RG14JJ 0RG14K0 0RG14K1 ///

0RG14KJ 0RG14Z0 0RG14Z1 0RG14ZJ 0RG40J0 0RG40J1 0RG40JJ 0RG40K0 0RG40K1 ///

0RG40KJ 0RG40Z0 0RG40Z1 0RG40ZJ 0RG43J0 0RG43J1 0RG43JJ 0RG43K0 0RG43K1 ///

0RG43KJ 0RG43Z0 0RG43Z1 0RG43ZJ 0RG44J0 0RG44J1 0RG44JJ 0RG44K0 0RG44K1 ///

0RG44KJ 0RG44Z0 0RG44Z1 0RG44ZJ 0RG60J0 0RG60J1 0RG60JJ 0RG60K0 0RG60K1 ///

0RG60KJ 0RG60Z0 0RG60Z1 0RG60ZJ 0RG63J0 0RG63J1 0RG63JJ 0RG63K0 0RG63K1 ///

0RG63KJ 0RG63Z0 0RG63Z1 0RG63ZJ 0RG64J0 0RG64J1 0RG64JJ 0RG64K0 0RG64K1 ///

0RG64KJ 0RG64Z0 0RG64Z1 0RG64ZJ 0RGA0J0 0RGA0J1 0RGA0JJ 0RGA0K0 0RGA0K1 ///

0RGA0KJ 0RGA0Z0 0RGA0Z1 0RGA0ZJ 0RGA3J0 0RGA3J1 0RGA3JJ 0RGA3K0 0RGA3K1 ///

0RGA3KJ 0RGA3Z0 0RGA3Z1 0RGA3ZJ 0RGA4J0 0RGA4J1 0RGA4JJ 0RGA4K0 0RGA4K1 ///

0RGA4KJ 0RGA4Z0 0RGA4Z1 0RGA4ZJ 0SG00J0 0SG00J1 0SG00JJ 0SG00K0 0SG00K1 ///

0SG00KJ 0SG00Z0 0SG00Z1 0SG00ZJ 0SG03J0 0SG03J1 0SG03JJ 0SG03K0 0SG03K1 ///

```
        0SG03KJ 0SG03Z0 0SG03Z1 0SG03ZJ 0SG04J0 0SG04J1 0SG04JJ 0SG04K0 0SG04K1 ///
        0SG04KJ 0SG04Z0 0SG04Z1 0SG04ZJ

        foreach td_z of local td_icd_7 {
                replace tech_dep_ccc = 1 if substr(`pc'`i',1,7)=="`td_z'"
                }

* TRANSPLANTATION CCC ICD-10

local tx_icd_7 02YA0Z0 02YA0Z1 02YA0Z2 0BYC0Z0 0BYC0Z1 0BYC0Z2 0BYD0Z0 0BYD0Z1 ///
        0BYD0Z2 0BYF0Z0 0BYF0Z1 0BYF0Z2 0BYG0Z0 0BYG0Z1 0BYG0Z2 0BYH0Z0 0BYH0Z1 ///
        0BYH0Z2 0BYJ0Z0 0BYJ0Z1 0BYJ0Z2 0BYK0Z0 0BYK0Z1 0BYK0Z2 0BYL0Z0 0BYL0Z1 ///
        0BYL0Z2 0BYM0Z0 0BYM0Z1 0BYM0Z2 0TY00Z0 0TY00Z1 0TY00Z2 0TY10Z0 0TY10Z1 ///
        0TY10Z2 0DY80Z0 0DY80Z1 0DY80Z2 0DYE0Z0 0DYE0Z1 0DYE0Z2 0FY00Z0 0FY00Z1 ///
        0FY00Z2 0FYG0Z0 0FYG0Z1 0FYG0Z2 3E030U0 3E030U1 3E033U0 3E033U1 3E0J3U0 ///
        3E0J3U1 3E0J7U0 3E0J7U1 3E0J8U0 3E0J8U1 07YP0Z0 07YP0Z1 07YP0Z2 30230AZ ///
        30230G0 30230G1 30230X0 30230X1 30230Y0 30230Y1 30233AZ 30233G0 30233G1 ///
        30233X0 30233X1 30233Y0 30233Y1 30240AZ 30240G0 30240G1 30240X0 30240X1 ///
        30240Y0 30240Y1 30243AZ 30243G0 30243G1 30243X0 30243X1 30243Y0 30243Y1 ///
        30250G0 30250G1 30250X0 30250X1 30250Y0 30250Y1 30253G0 30253G1 30253X0 ///
        30253X1 30253Y0 30253Y1 30260G0 30260G1 30260X0 30260X1 30260Y0 30260Y1 ///
        30263G0 30263G1 30263X0 30263X1 30263Y0 30263Y1

        foreach tx_z of local tx_icd_7 {
                replace transplant_ccc = 1 if substr(`pc'`i',1,7)=="`tx_z'"
                }
}
}
}
* Generate counts
gen num_ccc = 0
replace num_ccc = (neuromusc_ccc + cvd_ccc + respiratory_ccc + renal_ccc + ///
        gi_ccc + hemato_immu_ccc + metabolic_ccc + congeni_genetic_ccc + ///
        malignancy_ccc + neonatal_ccc)

* Generate CCC flag
```

```
gen ccc_flag = 0
replace ccc_flag = 1 if num_ccc>0
replace ccc_flag = 1 if tech_dep_ccc==1
replace ccc_flag = 1 if transplant_ccc==1

* Create variable labels
label variable ccc_flag "CCC: Any Presence of CCC"
label variable num_ccc "CCC: Number Recorded"
label variable neuromusc_ccc "CCC: Neuromuscular"
label variable cvd_ccc "CCC: Cardiovascular"
label variable respiratory_ccc "CCC: Respiratory"
label variable renal_ccc "CCC: Renal"
label variable gi_ccc "CCC: Gastrointestinal"
label variable hemato_immu_ccc "CCC: Heme/Immuno"
label variable metabolic_ccc "CCC: Metabolic"
label variable congeni_genetic_ccc "CCC: Congenital/Genetic"
label variable malignancy_ccc "CCC: Malignancy"
label variable neonatal_ccc "CCC: Neonatal"
label variable tech_dep_ccc "CCC: Technology Dependant"
label variable transplant_ccc "CCC: Transplant"

* Resort to original configuration
gsort +id
drop id dx_count pc_count

* Compress and save as new data file
compress
save `dt_out'_ccc, replace

end
exit
```
