# Supplementary material for: Pediatric complex chronic conditions classification system version 2: updated for ICD-10 and complex medical technology dependence and transplantation
Source: BMC Pediatr. 2014 Aug 8;14:199. doi: 10.1186/1471-2431-14-199 (PMC4134331; doi:10.1186/1471-2431-14-199)
Supplement: Additional file 5 — Categories of CCC v2 and the Corresponding ICD-9 and ICD-10 Diagnosis and Procedure Codes. Table of CCC categories and the corresponding ICD-9 and ICD-10 diagnosis and procedure codes. [file 1471-2431-14-199-S5.docx]

**Appendix Table: Categories of CCC v2 and the Corresponding ICD-9 and ICD-10 Diagnosis and Procedure Codes**

| **Categories** | **Subcategories** | **ICD-9** | **ICD-10** |
| --- | --- | --- | --- |
| **Neurologic and Neuromuscular** | Brain and spinal cord malformations | 740.0-742.9 | Q00-Q07, G90.1 |
|  | Mental retardation | 318.0-318.2 | F71-F73 |
|  | CNS degeneration and diseases | 330.0-330.9, 334, 335.0-335.9, 331.1, 331.11, 331.19, 331.4, 331.8, 331.89, 331.9, 333.2, 336.1, 336.8, 337.9, 759.5 | E75.0, E75.1, E75.2, E75.4, F84.2, G11.1-G11.4, G11.8, G11.9, G12.0- G12.2, G12.8, G12.9, G31.01, G31.09, G31.8, G31.89, G32.89, G93.8, G93.9, G94, G91.1, G31.9, G25.3, G95.19, G95.89, G90.9, Q85.1 |
|  | Infantile cerebral palsy | 343.0-343.9 | G80 |
|  | Epilepsy | 345.01, 345.11, 345.3, 345.41, 345.61, 345.71, 345.81, 345.91 | G40.311, G40.301, G40.211, G40.219, G40.411, G40.419, G40.111, G40.119, G40.804, G40.911, G40.919 |
|  | Other disorders of CNS | 341.8, 342.90, 344.0, 344.81, 344.9, 348.1, 348.4, 780.03, 01.52, 01.53 | G37.1, G37.2, G37.8, G81.90, G82.90, G82.50-G82.54, G83.5, G83.9, G93.1, G93.5, R40.3, 0016070, 0016071, 0016072, 0016073, 0016074, 0016075, 0016076, 0016077, 0016078, 001607B, 0016370, 0016371, 0016372, 0016373, 0016374, 0016375, 0016376, 0016377, 0016378, 001637B, 001U074, 001U076, 001U077, 001U079, 001U374, 001U376, 001U377, 001U379, 00B70ZZ, 00B73ZZ, 00B74ZZ, 00T70ZZ, 00T73ZZ, 00T74ZZ |
|  | Occlusion of cerebral arteries | 434.01, 434.91 | I63.30, I63.50 |
|  | Muscular dystrophies and myopathies | 359.0-359.3 | G71, G72 |
|  | Movement diseases | 332.0, 332.1, 333.0, 333.2,33.4, 333.5, 333.7, 333.9 | G10, G20, G21.0, G21.11, G21.19, G21.8, G23.0-G23.2, G23.8, G24.02, G24.8, G25.3-G25.5, G25.81-G25.83, G25.89, G25.9, G80.3 |
|  | Devices | 996.2, 996.63, V45.2, V53.01, V53.02, 02.2, 02.21, 02.22, 02.3, 02.31, 02.32, 02.33, 02.34, 02.35, 02.39, 02.4, 02.41, 02.42, 02.93, 03.7, 03.71, 03.72. 03.79, 03.93, 03.97, 04.92 | T85.09XA, T85.190A, T85.192A, T85.199A, T85.79XA, Z98.2, Z45.41, Z45.42, 00160J0, 00160J1, 00160J2, 00160J3, 00160J4, 00160J5, 00160J6, 00160J7, 00160J8, 00160JB, 00160K0, 00160K1, 00160K2, 00160K3, 00160K4, 00160K5, 00160K6, 00160K7, 00160K8, 00160KB, 00163J0, 00163J1, 00163J2, 00163J3, 00163J4, 00163J5, 00163J6, 00163J7, 00163J8, 00163JB, 00163K0, 00163K1, 00163K2, 00163K3, 00163K4, 00163K5, 00163K6, 00163K7, 00163K8, 00163KB, 001U0J4, 001U0J6, 001U0J7, 001U0J9, 001U0K4, 001U0K6, 001U0K7, 001U0K9, 001U3J4, 001U3J6, 001U3J7, 001U3J9, 001U3K4, 001U3K6, 001U3K7, 001U3K9, 009600Z, 009630Z, 009640Z, 00H00MZ, 00H03MZ, 00H04MZ, 00H60MZ, 00H63MZ, 00H64MZ, 00HE0MZ, 00HE3MZ, 00HE4MZ, 00HU0MZ, 00HU3MZ, 00HU4MZ, 00HV0MZ, 00HV3MZ, 00HV4MZ, 00W60JZ, 00W63JZ, 00W64JZ, 00WU0JZ, 00WU3JZ, 00WU4JZ, 01HY0MZ, 01HY3MZ, 01HY4MZ, 0DH60MZ, 0DH63MZ, 0DH64MZ, 0W110J9, 0W110JB, 0W110JG, 0W110JJ, 3E1Q38X, 3E1Q38Z |
|  | Transplantation | N/A | N/A |
|  | | | |
| **Cardiovascular** | Heart and great vessel malformations | 745.0-745.3, 745.60-745.69, 746, 747.1-747.49, 747.81, 747.89, 35.8, 35.81, 35.82, 35.83, 35.84 | Q20, Q21.2-Q24, Q25.1-Q26, Q28.2, Q28.3, Q28.9, 02170ZP, 02170ZQ, 02170ZR, 02BK0ZZ, 02LR0ZT, 02LS0ZZ, 02LT0ZZ, 02NH0ZZ, 02RK0JZ, 02RL0JZ, 02RM0JZ, 02RP0JZ, 02RQ07Z, 02RQ0JZ, 02RR07Z, 02RR0JZ, 02SP0ZZ, 02SW0ZZ, 02U70JZ, 02UA0JZ, 02UA3JZ, 02UA4JZ, 02VR0ZT, 02WA0JZ |
|  | Endocardium diseases | 424.0, 424.2, 424.3 | I34.0, I34.8, I36.0, I36.8, I37.0, I37.8 |
|  | Cardiomyopathies | 425.0-425.4, 425.8, 429.1 | I42, I43, I51.5 |
|  | Conduction disorder | 426.0-427.4 | I44, I45, I47, I48, I49.0 |
|  | Dysrhythmias | 427.6-427.9 | I49.1-I49.5, I49.8, I49.9, R00.1 |
|  | Other | 416.1, 416.8, 416.9, 428.0, 429.3, 428.83,  433.11, V45.81 | I27.0, I27.1, I27.2, I27.81, I27.89, I27.9, I50.9, I51.7, I51.81, I63.139, I63.239, Z95.1 |
|  | Devices | 996.0, 996.1, 996.61, 996.62, V43.3, V45.0, V53.31, V53.32, V53.39, 00.50, 00.51, 00.53, 00.54, 00.55, 00.57, 17.51, 17.52, 37.41, 37.52, 37.53, 37.54, 37.55, 37.6, 37.60, 37.61, 37.63, 37.65, 37.66, 37.67, 37.68, 37.7, 37.71, 37.72, 37.74, 37.76, 37.79, 37.8, 37.80, 37.81, 37.82, 37.83, 37.85, 37.86, 37.87, 37.89, 37.94, 37.95, 37.96, 37.97, 37.98, 39.81, 39.82, 39.83, 39.84, 39.85, 89.46, 89.47, 89.48, 89.49 | T82.519A, T82.529A, T82.539A, T82.599A, T82.110A, T82.111A, T82.120A, T82.121A, T82.190A, T82.191A, T82.01XA, T82.02XA, T82.03XA, T82.09XA, T82.211A, T82.212A, T82.213A, T82.218A, T82.221A, T82.222A, T82.223A, T82.228A, T82.518A, T82.528A, T82.538A, T82.598A, T82.6XXA, T82.7XXA, Z95.0, Z95.2, Z95.3, Z95.810-Z95.812, Z95.818, Z45.010, Z45.018, Z45.02, Z45.09, Z95.9, 02H40JZ, 02H40KZ, 02H43JZ, 02H44JZ, 02H44KZ, 02H60JZ, 02H60KZ, 02H63JZ, 02H63KZ, 02H63MZ, 02H64JZ, 02H64KZ, 02H70KZ, 02H73JZ, 02H73KZ, 02H73MZ, 02H74KZ, 02HA0QZ, 02HA0RS, 02HA0RZ, 02HA3QZ, 02HA3RS, 02HA4QZ, 02HA4RS, 02HK0JZ, 02HK0KZ, 02HK3JZ, 02HK3KZ, 02HK3MZ, 02HK4JZ, 02HK4KZ, 02HL0JZ, 02HL0KZ, 02HL0MZ, 02HL3JZ, 02HL3KZ, 02HL3MZ, 02HL4JZ, 02HL4KZ, 02HL4MZ, 02HN0JZ, 02HN0KZ, 02HN0MZ, 02HN3JZ, 02HN3KZ, 02HN3MZ, 02HN4JZ, 02HN4KZ, 02HN4MZ, 02WA0QZ, 02WA0RZ, 02WA3QZ, 02WA3RZ, 02WA4QZ, 02WA4RZ, 03HK0MZ, 03HK3MZ, 03HK4MZ, 03HL0MZ, 03HL3MZ, 03HL4MZ, 03WY0MZ, 03WY3MZ, 03WY4MZ, 0JH600Z, 0JH605Z, 0JH606Z, 0JH607Z, 0JH608Z, 0JH609Z, 0JH60AZ, 0JH60MZ, 0JH60PZ, 0JH630Z, 0JH635Z, 0JH636Z, 0JH637Z, 0JH638Z, 0JH639Z, 0JH63AZ, 0JH63MZ, 0JH63PZ, 0JH70MZ, 0JH73MZ, 0JH800Z, 0JH805Z, 0JH806Z, 0JH807Z, 0JH808Z, 0JH809Z, 0JH80AZ, 0JH80MZ, 0JH80PZ, 0JH830Z, 0JH835Z, 0JH836Z, 0JH837Z, 0JH838Z, 0JH839Z, 0JH83AZ, 0JH83MZ, 0JH83PZ, 0JWT0MZ, 0JWT0PZ, 0JWT3MZ, 0JWT3PZ, 0JWTXMZ, 4B02XSZ, 4B02XTZ, 5A02110, 5A02116, 5A0211D, 5A02210, 5A02216, 5A0221D |
|  | Transplantation | 996.83,V42.1, V42.2, V43.2, 37.5, 37.51 | T86.20-T86.22, Z94.1, 02YA0Z0, 02YA0Z1, 02YA0Z2 |
|  | | | |
| **Respiratory** | Respiratory malformations | 748.0-748.9 | Q30-Q34, P280 |
|  | Chronic respiratory diseases | 327.25, 416.2, 516.3, 516.31, 518.84, 770.4, V45.76 | G47.35, I27.82, I43, J84.112, J96.20, Z90.2 |
|  | Cystic fibrosis | 277.0 | E84 |
|  | Other | 30.3, 30.4, 32.4, 32.41, 32.49, 32.5, 32.50, 32.59 | 0B110Z4, 0B113Z4, 0B114Z4, 0BTC0ZZ, 0BTC4ZZ, 0BTD0ZZ, 0BTD4ZZ, 0BTF0ZZ, 0BTF4ZZ, 0BTG0ZZ, 0BTG4ZZ, 0BTJ0ZZ, 0BTJ4ZZ, 0BTK0ZZ, 0BTK4ZZ, 0BTL0ZZ, 0BTL4ZZ, 0BTM0ZZ, 0BTM4ZZ, 0CTS0ZZ, 0CTS4ZZ, 0CTS7ZZ, 0CTS8ZZ |
|  | Devices | 519.0, V44.0, V55.0, V46.0, V46.1, 31.2, 31.21, 31.29, 31.41, 31.74, 33.21, 34.85, 96.55, 97.23 | J95.00-J95.04, J95.09, Z43.0, Z93.0, Z99.0, J95.850, Z99.11, Z99.12, 0B110F4, 0B113F4, 0B114F4, 0B21XFZ, 0BHR0MZ, 0BHR3MZ, 0BHR4MZ, 0BHS0MZ, 0BHS3MZ, 0BHS4MZ, 0BW10FZ, 0BW13FZ, 0BW14FZ, 0JH604Z, 0JH634Z, 0JH804Z, 0JH834Z, 0WQ6XZ2, 3E1F78Z |
|  | Transplantation | 996.84,V42.6, 33.5, 33.50, 33.51, 33.52, 33.6 | T86.810, T86.811, T86.819, Z94.2, 0BYC0Z0, 0BYC0Z1, 0BYC0Z2, 0BYD0Z0, 0BYD0Z1, 0BYD0Z2, 0BYF0Z0, 0BYF0Z1, 0BYF0Z2, 0BYG0Z0, 0BYG0Z1, 0BYG0Z2, 0BYH0Z0, 0BYH0Z1, 0BYH0Z2, 0BYJ0Z0, 0BYJ0Z1, 0BYJ0Z2, 0BYK0Z0, 0BYK0Z1, 0BYK0Z2, 0BYL0Z0, 0BYL0Z1, 0BYL0Z2, 0BYM0Z0, 0BYM0Z1, 0BYM0Z2 |
|  | | | |
| **Renal and Urologic** | Congenital anomalies | 753.0-753.9 | Q60-Q64 |
|  | Chronic renal failure | 585 | N18 |
|  | Other | V45.73, V45.74, 55.5, 55.51, 55.52, 55.53, 55.54, 56.4, 56.41, 56.42, 56.7, 56.71, 56.79, 57.7, 57.71, 57.79 | Z90.5, Z90.6, 0T160Z8, 0T160ZA, 0T164Z8, 0T164ZA, 0T170Z8, 0T170ZA, 0T174Z8, 0T174ZA, 0T180Z8, 0T180ZA, 0T184Z8, 0T184ZA, 0TB60ZZ, 0TB63ZZ, 0TB64ZZ, 0TB67ZZ, 0TB68ZZ, 0TB70ZZ, 0TB73ZZ, 0TB74ZZ, 0TB77ZZ, 0TB78ZZ, 0TT00ZZ, 0TT04ZZ, 0TT10ZZ, 0TT14ZZ, 0TT20ZZ, 0TT24ZZ, 0TT60ZZ, 0TT64ZZ, 0TT67ZZ, 0TT68ZZ, 0TT70ZZ, 0TT74ZZ, 0TT77ZZ, 0TT78ZZ, 0TTB0ZZ, 0TTB4ZZ, 0TTB7ZZ, 0TTB8ZZ, 0TTD0ZZ, 0TTD4ZZ, 0TTD7ZZ, 0TTD8ZZ |
|  | Chronic bladder diseases | 344.61, 596.4, 596.53, 596.54 | G83.4, N31.2, N31.9 |
|  | Devices | 996.68, V44.5, V44.6, V45.1, V53.6, V55.5, V55.6, 38.95, 39.27, 39.42, 39.93, 39.94, 39.95, 54.98, 55.02, 55.03, 55.04, 55.12, 55.93, 55.94, 55.97, 56.5, 56.51, 56.52, 56.6, 56.61, 56.62, 56.72, 56.73, 56.74, 56.75, 57.2, 57.21, 57.22, 59.93, 59.94, 86.07, 96.45, 96.46, 96.47 | T85.71XA, Z93.50-Z93.52, Z93.59, Z93.6, Z91.15, Z99.2, Z43.5, Z43.6, Z46.6, 031209D, 031209F, 03120AD, 03120AF, 03120JD, 03120JF, 03120KD, 03120KF, 03120ZD, 03120ZF, 031309D, 031309F, 03130AD, 03130AF, 03130JD, 03130JF, 03130KD, 03130KF, 03130ZD, 03130ZF, 031409D, 031409F, 03140AD, 03140AF, 03140JD, 03140JF, 03140KD, 03140KF, 03140ZD, 03140ZF, 031509D, 031509F, 03150AD, 03150AF, 03150JD, 03150JF, 03150KD, 03150KF, 03150ZD, 03150ZF, 031609D, 031609F, 03160AD, 03160AF, 03160JD, 03160JF, 03160KD, 03160KF, 03160ZD, 03160ZF, 031709D, 031709F, 03170AD, 03170AF, 03170JD, 03170JF, 03170KD, 03170KF, 03170ZD, 03170ZF, 031809D, 031809F, 03180AD, 03180AF, 03180JD, 03180JF, 03180KD, 03180KF, 03180ZD, 03180ZF, 031909F, 03190AF, 03190JF, 03190KF, 03190ZF, 031A09F, 031A0AF, 031A0JF, 031A0KF, 031A0ZF, 031B09F, 031B0AF, 031B0JF, 031B0KF, 031B0ZF, 031C09F, 031C0AF, 031C0JF, 031C0KF, 031C0ZF, 03WY0JZ, 03WY3JZ, 03WY4JZ, 03WYXJZ, 05HY33Z, 06HY33Z, 0JH60WZ, 0JH60XZ, 0JH63WZ, 0JH63XZ, 0JH80WZ, 0JH80XZ, 0JH83WZ, 0JH83XZ, 0JHD0WZ, 0JHD0XZ, 0JHD3WZ, 0JHD3XZ, 0JHF0WZ, 0JHF0XZ, 0JHF3WZ, 0JHF3XZ, 0JHL0WZ, 0JHL0XZ, 0JHL3WZ, 0JHL3XZ, 0JHM0WZ, 0JHM0XZ, 0JHM3WZ, 0JHM3XZ, 0T130ZB, 0T134ZB, 0T140ZB, 0T144ZB, 0T16079, 0T1607C, 0T1607D, 0T160J9, 0T160JC, 0T160JD, 0T160K9, 0T160KC, 0T160KD, 0T160Z9, 0T160ZC, 0T160ZD, 0T163JD, 0T16479, 0T1647C, 0T1647D, 0T164J9, 0T164JC, 0T164JD, 0T164K9, 0T164KC, 0T164KD, 0T164Z9, 0T164ZC, 0T164ZD, 0T17079, 0T1707C, 0T1707D, 0T170J9, 0T170JC, 0T170JD, 0T170K9, 0T170KC, 0T170KD, 0T170Z9, 0T170ZC, 0T170ZD, 0T173JD, 0T17479, 0T1747C, 0T1747D, 0T174J9, 0T174JC, 0T174JD, 0T174K9, 0T174KC, 0T174KD, 0T174Z9, 0T174ZC, 0T174ZD, 0T18079, 0T1807C, 0T1807D, 0T180J9, 0T180JC, 0T180JD, 0T180K9, 0T180KC, 0T180KD, 0T180Z9, 0T180ZC, 0T180ZD, 0T183JD, 0T18479, 0T1847C, 0T1847D, 0T184J9, 0T184JC, 0T184JD, 0T184K9, 0T184KC, 0T184KD, 0T184Z9, 0T184ZC, 0T184ZD, 0T1B0ZD, 0T1B4ZD, 0T25X0Z, 0T29X0Z, 0T29XYZ, 0T2BX0Z, 0T9000Z, 0T9030Z, 0T9040Z, 0T9070Z, 0T9080Z, 0T9100Z, 0T9130Z, 0T9140Z, 0T9170Z, 0T9180Z, 0T9370Z, 0T9380Z, 0T9470Z, 0T9480Z, 0TQ67ZZ, 0TQ77ZZ, 3E1K38Z, 3E1M39Z, 5A1D60Z |
|  | Transplantation | 996.81,V42.0, 55.6, 55.61, 55.69 | T86.10-T86.12, Z94.0, 0TY00Z0, 0TY00Z1, 0TY00Z2, 0TY10Z0, 0TY10Z1, 0TY10Z2 |
|  | | | |
| **Gastrointestinal** | Congenital anomalies | 750.3, 751.1-751.9 | Q39.0-Q39.4, Q41-Q45 |
|  | Chronic liver disease and cirrhosis | 571.4-571.9 | K73, K74, K75.4, K760-K763, K765, K768 |
|  | Inflammatory bowel diseases | 555.0-556.9 | K50, K51 |
|  | Other | 453.0, 557.1, 560.2, 564.7, V45.3, V45.72, V45.75, 25.3, 25.4, 42.42, 43.9, 43.91, 43.99, 45.63, 45.8, 45.81, 45.82, 45.83, 50.4, 52.6, 52.7, 54.71 | I82.0, K55.1, K56.2, K59.3, Z98.0, Z90.3, Z90.49, 0CT70ZZ, 0CT7XZZ, 0D13079, 0D1307A, 0D1307B, 0D1607A, 0D160ZA, 0DT50ZZ, 0DT54ZZ, 0DT57ZZ, 0DT58ZZ, 0DT60ZZ, 0DT64ZZ, 0DT67ZZ, 0DT68ZZ, 0DT80ZZ, 0DT84ZZ, 0DT87ZZ, 0DT88ZZ, 0DT90ZZ, 0DT94ZZ, 0DT97ZZ, 0DT98ZZ, 0DTE0ZZ, 0DTE4ZZ, 0DTE7ZZ, 0DTE8ZZ, 0FT00ZZ, 0FT04ZZ, 0FTG0ZZ, 0FTG4ZZ |
|  | Devices | 536.4, V44.1-V44.4, V53.50, V53.51, V53.59, V55.1-V55.4, 42.1, 42.10, 42.11, 42.81, 43.1, 43.11, 43.19, 44.12, 44.3, 44.32, 44.38, 44.39, 46.1, 46.13, 46.2, 46.22, 46.23, 46.3, 46.32, 46.4, 46.40, 46.41, 46.43, 96.24, 96.36, 97.02 | K94.20, K94.22, K94.23, K94.29, Z93.1-Z93.4, Z43.1-Z43.4, Z46.51, Z46.59, 0D11074, 0D110J4, 0D110K4, 0D110Z4, 0D113J4, 0D11474, 0D114J4, 0D114K4, 0D114Z4, 0D15074, 0D150J4, 0D150K4, 0D150Z4, 0D153J4, 0D15474, 0D154J4, 0D154K4, 0D154Z4, 0D16074, 0D160J4, 0D160J9, 0D160JA, 0D160K4, 0D160K9, 0D160KA, 0D160Z4, 0D163J4, 0D16474, 0D164J4, 0D164J9, 0D164JA, 0D164K4, 0D164K9, 0D164KA, 0D164Z4, 0D16874, 0D168J4, 0D168J9, 0D168JA, 0D168K4, 0D168K9, 0D168KA, 0D168Z4, 0D1B0Z4, 0D1B4Z4, 0D1B8Z4, 0D1H0Z4, 0D1H4Z4, 0D1H8Z4, 0D1K0Z4, 0D1K4Z4, 0D1K8Z4, 0D1L0Z4, 0D1L4Z4, 0D1L8Z4, 0D1N0Z4, 0D1N4Z4, 0D1N8Z4, 0D20X0Z, 0D20XUZ, 0D20XYZ, 0D787ZZ, 0D7E7ZZ, 0DBB7ZZ, 0DH50DZ, 0DH50UZ, 0DH53DZ, 0DH53UZ, 0DH54DZ, 0DH54UZ, 0DH57DZ, 0DH57UZ, 0DH58DZ, 0DH58UZ, 0DH63UZ, 0DH64UZ, 0DHA3UZ, 0DHA4UZ, 0DHA8UZ, 0DN87ZZ, 0DNE7ZZ, 0DW04UZ, 0DW08UZ, 0WQFXZ2, 3E1G78Z, 3E1H78Z |
|  | Transplantation | 996.82,996.86,996.87,V42.7, V42.83, V42.84, 46.97, 50.5, 50.51, 50.59, 52.8, 52.80, 52.82, 52.83, 52.84, 52.85, 52.86 | T86.40-T86.42, T86.890, T86.891, T86.899, T86.850, T86.851, T86.859, Z94.4, Z94.82, Z94.83, 0DY80Z0, 0DY80Z1, 0DY80Z2, 0DYE0Z0, 0DYE0Z1, 0DYE0Z2, 0FY00Z0, 0FY00Z1, 0FY00Z2, 0FYG0Z0, 0FYG0Z1, 0FYG0Z2, 3E030U0, 3E030U1, 3E033U0, 3E033U1, 3E0J3U0, 3E0J3U1, 3E0J7U0, 3E0J7U1, 3E0J8U0, 3E0J8U1 |
|  | | | |
| **Hematologic or immunologic** | Hereditary anemias | 282.0-282.6 | D55-D58 |
|  | Aplastic anemias | 284 | D60-D61, D71 |
|  | Hereditary immunodeficiency | 279.0-279.9, 288.1, 288.2, 446.1 | D80-D89, D72.0, M30.3, M35.9 |
|  | Coagulation/hemorrhagic | 286.0, 286.3, 287.32, 287.33, 287.39 | D66, D68.2, D69.41, D69.42, D69.49 |
|  | Leukopenia | 288.01, 288.02 | D70.0, D70.4 |
|  | Hemophagocytic Syndromes | 288.4 | D76.1-D76.3 |
|  | Sarcoidosis | 135 | D86.9 |
|  | Acquired immunodeficiency | 042-044 | B20-B24 |
|  | Polyarteritis nodosa and related conditions | 446.0, 446.21, 446.4-446.7 | M30.0, M31.0, M31.1, M31.30, M31.4, M31.6 |
|  | Diffuse diseases of connective tissue | 710.0, 710.1, 710.3 | M32.10, M33.90, M34.0, M34.1, M34.9 |
|  | Other | 41.5 | 07TP0ZZ, 07TP4ZZ |
|  | Devices | N/A | N/A |
|  | Transplantation | 41.00, 41.01, 41.02, 41.03, 41.04, 41.05, 41.06, 41.07, 41.08, 41.09, 41.94 | 07YP0Z0, 07YP0Z1, 07YP0Z2, 30230AZ, 30230G0, 30230G1, 30230X0, 30230X1, 30230Y0, 30230Y1, 30233AZ, 30233G0, 30233G1, 30233X0, 30233X1, 30233Y0, 30233Y1, 30240AZ, 30240G0, 30240G1, 30240X0, 30240X1, 30240Y0, 30240Y1, 30243AZ, 30243G0, 30243G1, 30243X0, 30243X1, 30243Y0, 30243Y1, 30250G0, 30250G1, 30250X0, 30250X1, 30250Y0, 30250Y1, 30253G0, 30253G1, 30253X0, 30253X1, 30253Y0, 30253Y1, 30260G0, 30260G1, 30260X0, 30260X1, 30260Y0, 30260Y1, 30263G0, 30263G1, 30263X0, 30263X1, 30263Y0, 30263Y1 |
|  | | | |
| **Metabolic** | Amino acid metabolism | 270.0-270.9 | E70.0, E70.2, E70.3, E70.4, E70.8, E71.0-E71.5, E72.0-E72.4, E72.8, E72.9 |
|  | Carbohydrate metabolism | 271.0-271.9 | E74.0-E74.4, E74.8, E74.9 |
|  | Lipid metabolism | 272.0-272.9 | E75, E77.0, E77.1, E78.0-E78.4, E78.5-E78.9, E88.1, E88.8 |
|  | Storage disorder | 277.3, 277.5 | E76.0-E76.3, E85 |
|  | Other metabolic disorders | 275.0-275.3, 277.2,277.6, 277.8-277.9 | 277.4, E79.1, E79.8, E80.4-E80.7, E83.0, E83.1, E83.3, E83.4, D84.1, E88, H49.8 |
|  | Endocrine disorders | 243, 253.2, 253.5, 253.6, 235.9, 255.0, 255.13, 255.2, 06.4, 06.52, 06.81, 07.3, 07.64, 07.65, 07.68, 07.69, 62.4, 62.41, 64.5, 65.5, 65.51, 65.53, 65.6, 65.61, 65.63, 68.4, 68.41, 68.49, 68.5, 68.51, 68.59, 68.6, 68.61, 68.69, 68.7, 68.71, 68.79 | E00.9, E23.0, E23.2, E22.2, E23.3, E23.7, E24.0, E24.2, E24.3, E24.8, E24.9, E26.81, E25.0, E25.8, E25.9, 0GT00ZZ, 0GT04ZZ, 0GT40ZZ, 0GT44ZZ, 0GTK0ZZ, 0GTK4ZZ, 0GTR0ZZ, 0GTR4ZZ, 0UT20ZZ, 0UT24ZZ, 0UT27ZZ, 0UT28ZZ, 0UT2FZZ, 0UT40ZZ, 0UT44ZZ, 0UT47ZZ, 0UT48ZZ, 0UT70ZZ, 0UT74ZZ, 0UT90ZZ, 0UT94ZZ, 0UT97ZZ, 0UT98ZZ, 0UT9FZZ, 0UTC0ZZ, 0UTC7ZZ, 0UTC8ZZ, 0VTC0ZZ, 0VTC4ZZ, 0W4M070, 0W4M0J0, 0W4M0K0, 0W4M0Z0, 0W4N071, 0W4N0J1, 0W4N0K1, 0W4N0Z1 |
|  | Devices | V45.85, V53.91, 86.06 | Z46.81, Z96.41, 0JH60VZ, 0JH63VZ, 0JH70VZ, 0JH73VZ, 0JH80VZ, 0JH83VZ, 0JHD0VZ, 0JHD3VZ, 0JHF0VZ, 0JHF3VZ, 0JHG0VZ, 0JHG3VZ, 0JHH0VZ, 0JHH3VZ, 0JHL0VZ, 0JHL3VZ, 0JHM0VZ, 0JHM3VZ, 0JHN0VZ, 0JHN3VZ, 0JHP0VZ, 0JHP3VZ, 0JHT0VZ, 0JHT3VZ |
|  | Transplantation | N/A | N/A |
|  | | | |
| **Other Congenital or Genetic Defect** | Chromosomal anomalies | 758.0-758.9 | Q90.9, Q91.3, Q91.4, Q91.7, Q92.8, Q93, Q95.0, Q96.9, Q97, Q98, Q99.8, Q99.9 |
|  | Bone and join anomalies | 259.4, 737.3, 756.0-756.5 | E34.3, M41.0, M41.2, M41.30, M41.8, M41.9, M43.30, M96.5, Q72.2, Q75.0, Q75.2, Q75.9, Q76.0-Q76.2, Q76.4-Q76.7, Q77, Q78.0-Q78.4, Q78.8, Q78.9 |
|  | Diaphragm and abdominal wall | 553.3, 756.6, 756.7 | K44.9, Q79.0-Q79.5, Q79.9, Q79.59 |
|  | Other congenital anomalies | 757.39, 759.7-759.9 | Q81, Q87.1-Q87.3, Q87.40, Q87.81, Q87.89, Q89.7, Q89.9, Q99.2 |
|  | | | |
| **Malignancy** | Neoplasms | 140-209, 230-239, 00.10, 99.25 | C00-C96, D01-D09, D3A.0, D37-D49, Q85.0, 3E00X05, 3E01305, 3E02305, 3E03005, 3E03305, 3E04005, 3E04305, 3E05005, 3E05305, 3E06005, 3E06305, 3E0A305, 3E0F305, 3E0F705, 3E0F805, 3E0G305, 3E0G705, 3E0G805, 3E0H305, 3E0H705, 3E0H805, 3E0J305, 3E0J705, 3E0J805, 3E0K305, 3E0K705, 3E0K805, 3E0L305, 3E0L705, 3E0M305, 3E0M705, 3E0N305, 3E0N705, 3E0N805, 3E0P305, 3E0P705, 3E0P805, 3E0Q305, 3E0Q705, 3E0R305, 3E0S305, 3E0V305, 3E0W305, 3E0Y305, 3E0Y705 |
|  | Devices | N/A | N/A |
|  | Transplantation | 996.85,V42.81, V42.82 | T86.00-T86.02, T86.09, Z94.81, Z94.84 |
|  | | | |
| **Premature and Neonatal** | Fetal malnutrition | 764.01, 764.02, 764.11, 764.12, 764.21, 764.22, 764.91, 764.92 | P05.01, P05.11, P05.02, P05.12, P05.2, P05.9 |
|  | Extreme immaturity | 765.01, 765.02, 765.11, 765.12, 765.21-765.23 | P07.01, P07.02, P07.21-P07.25 |
|  | Cerebral hemorrhage at birth | 767.0 | P10.0, P10.1, P10.4, P52.4, P52.8 |
|  | Spinal cord injury at birth | 767.4 | P11.5 |
|  | Birth asphyxia | 768.5, 768.9 | P21.0, P21.9, P84 |
|  | Respiratory diseases | 770.2, 770.7 | P25.0-P25.3, P25.8, P27.0, P27.1, P27.8 |
|  | Hypoxic-ischemic encephalopathy | 768.7 | P91.6 |
|  | Other | 771.0, 771.1, 772.13, 772.14, 773.3, 773.4, 774.7, 776.5, 777.53, 778.0, 779.7 | P35.0, P35.1, P25.21, P25.22, P56.0, P57.0, P57.8, P61.3, P61.4, P77.3, P83.2, P91.2 |
|  | | | |
| **Miscellaneous, Not Elsewhere Classified** | Devices | 996.4, 996.66, 996.67, 996.9, V46.2 81.00, 81.01, 81.02, 81.03, 81.04, 81.05, 81.06, 81.07, 81.08, 81.09, 81.30, 81.31, 81.32, 81.33, 81.34, 81.35, 81.36, 81.37, 81.38, 81.39, 84.51 | T84.019A, T84.029A, T84.039A, T84.049A, T84.059A, T84.069A, T84.099A, T84.498A, T84.119A, T84.129A, T84.199A, T84.498A, T84.50XA, T84.60XA, , T84.7XXA, T86.90-T86.92, T86.99, T86.10-T86.12, T86.40-T86.42, T86.20-T86.22, T86.810, T86.811, T86.819, T86.00-T86.02, T86.09, T86.890, T86.891, T86.899, T86.850, T86.851, T86.859, T86.5, T86.890, T86.891, T86.899, T87.0X9, T87.1X9, T87.2, Y83.1, Y83.3, Z99.81, 0RG00J0, 0RG00J1, 0RG00JJ, 0RG00K0, 0RG00K1, 0RG00KJ, 0RG00Z0, 0RG00Z1, 0RG00ZJ, 0RG03J0, 0RG03J1, 0RG03JJ, 0RG03K0, 0RG03K1, 0RG03KJ, 0RG03Z0, 0RG03Z1, 0RG03ZJ, 0RG04J0, 0RG04J1, 0RG04JJ, 0RG04K0, 0RG04K1, 0RG04KJ, 0RG04Z0, 0RG04Z1, 0RG04ZJ, 0RG10J0, 0RG10J1, 0RG10JJ, 0RG10K0, 0RG10K1, 0RG10KJ, 0RG10Z0, 0RG10Z1, 0RG10ZJ, 0RG13J0, 0RG13J1, 0RG13JJ, 0RG13K0, 0RG13K1, 0RG13KJ, 0RG13Z0, 0RG13Z1, 0RG13ZJ, 0RG14J0, 0RG14J1, 0RG14JJ, 0RG14K0, 0RG14K1, 0RG14KJ, 0RG14Z0, 0RG14Z1, 0RG14ZJ, 0RG40J0, 0RG40J1, 0RG40JJ, 0RG40K0, 0RG40K1, 0RG40KJ, 0RG40Z0, 0RG40Z1, 0RG40ZJ, 0RG43J0, 0RG43J1, 0RG43JJ, 0RG43K0, 0RG43K1, 0RG43KJ, 0RG43Z0, 0RG43Z1, 0RG43ZJ, 0RG44J0, 0RG44J1, 0RG44JJ, 0RG44K0, 0RG44K1, 0RG44KJ, 0RG44Z0, 0RG44Z1, 0RG44ZJ, 0RG60J0, 0RG60J1, 0RG60JJ, 0RG60K0, 0RG60K1, 0RG60KJ, 0RG60Z0, 0RG60Z1, 0RG60ZJ, 0RG63J0, 0RG63J1, 0RG63JJ, 0RG63K0, 0RG63K1, 0RG63KJ, 0RG63Z0, 0RG63Z1, 0RG63ZJ, 0RG64J0, 0RG64J1, 0RG64JJ, 0RG64K0, 0RG64K1, 0RG64KJ, 0RG64Z0, 0RG64Z1, 0RG64ZJ, 0RGA0J0, 0RGA0J1, 0RGA0JJ, 0RGA0K0, 0RGA0K1, 0RGA0KJ, 0RGA0Z0, 0RGA0Z1, 0RGA0ZJ, 0RGA3J0, 0RGA3J1, 0RGA3JJ, 0RGA3K0, 0RGA3K1, 0RGA3KJ, 0RGA3Z0, 0RGA3Z1, 0RGA3ZJ, 0RGA4J0, 0RGA4J1, 0RGA4JJ, 0RGA4K0, 0RGA4K1, 0RGA4KJ, 0RGA4Z0, 0RGA4Z1, 0RGA4ZJ, 0SG00J0, 0SG00J1, 0SG00JJ, 0SG00K0, 0SG00K1, 0SG00KJ, 0SG00Z0, 0SG00Z1, 0SG00ZJ, 0SG03J0, 0SG03J1, 0SG03JJ, 0SG03K0, 0SG03K1, 0SG03KJ, 0SG03Z0, 0SG03Z1, 0SG03ZJ, 0SG04J0, 0SG04J1, 0SG04JJ, 0SG04K0, 0SG04K1, 0SG04KJ, 0SG04Z0, 0SG04Z1, 0SG04ZJ |
|  | Transplantation | 996.80, 6.88, 996.89, 00.91, 00.92, 00.93 | T86.5, T86.90-T86.92, T86.99, T86.890, T86.891, T86.899 |
